# Supplementary material for: Cost-effectiveness of mechanical thrombectomy within 6 hours of acute ischaemic stroke in China
Source: BMJ Open. 2018 Feb 22;8(2):e018951. doi: 10.1136/bmjopen-2017-018951 (PMC5855394; doi:10.1136/bmjopen-2017-018951)

ONLINE SUPPLEMENTARY MATERIALS

Online supplementary table 1. Hypothetical worse performance and costs of mechanical thrombectomy, ICER (CNY/QALY)

	Outcome of mechanical thrombectomy		
**Additional cost of mechanical thrombectomy**	**Base Case (OR=2.046)***	**Unfavourable (OR=1.745)**	**Worse unfavourable (OR=1.692)**
+10%, CNY 66903	69,421	90,340	95,839
Base case, CNY 60821	63,010	82,120	87,123
-10%, CNY 54739	56,612	73,883	78,425
-25%, CNY 45616	47,003	61,553	65,360
-50%, CNY 30410	30,995	40,987	43,596

ICER, incremental cost-effectiveness ratio; QALY, quality-adjusted life-year; OR, Odds ratio.

* OR: OR of modified Rankin Scale 0-2 at day 90 for mechanical thrombectomy.

Online supplementary table 2. Distributions and parameters used in model inputs

**Model Input**	Distribution	α	β	mean	standard error
**Efficacy and safety outcome inputs**					
Proportion of outcomes at 90 days in IV tPA group					
mRS 0-2	beta	60.7	126.0		
Death (mRS 6)	beta	65.7	325.5		
sICH	beta	13.5	218.7		
Odds ratio at 90 days					
mRS 0-2	lognormal*			2.046	0.097
Death (mRS 6)	lognormal*			0.871	0.123
sICH	lognormal*			0.965	0.190
**Probabilities inputs**					
Proportion of patients received mechanical thrombectomy	beta	817.0	131.9		
Proportion of patients arrived within 4.5 hours	beta	1817.2	310.7		
Recurrent rate of stroke (per patient year)					
mRS 0-2	beta	833.0	7286.0		
mRS 3-5	beta	496.8	3006.6		
Relative risk of stroke recurrence per life year	lognormal*			1.03	0.005
Death with recurrent stroke	beta	290.9	1093.7		
Hazard ratio of non-stroke death for mRS 3-5	lognormal*			1.78	0.103
**Cost inputs (2013 Chinese Yuan Renminbi)**					
Additional costs of mechanical thrombectomy	lognormal			60821	4594
Additional costs of IV tPA treatment	lognormal			11179	325
Additional costs of sICH	lognormal			2374	65
One-time hospitalisation costs					
mRS 0-2	lognormal			10055	76
mRS 3-5	lognormal			13729	155
mRS 6	lognormal			11121	475
Annual post-hospitalisation costs					
mRS 0-2	lognormal			7385	118
mRS 3-5	lognormal			11350	323
**Utility inputs**					
mRS 0-2	beta	125.3	39.6		
mRS 3-5	beta	65.9	247.8		
Recurrent stroke	beta	732.4	1421.7		
sICH	beta	21.3	4.1		
**Discount rate inputs**					
Costs	beta	5.3	172.5		
Outcomes	beta	93.1	3011.2		

IV tPA, intravenous tissue plasminogen activator; mRS, modified Rankin Scale; sICH, symptomatic intracerebral haemorrhage.

*Correction is implemented in the Ersatz function ErRelativeRisk(RR, SE[ln(RR)]).

Online supplementary figure 1. Cost-effectiveness acceptability curve. The curve presents the probability that mechanical thrombectomy within 6 hours to be cost-effective against willingness-to-pay threshold. The solid line represents the willingness-to-pay threshold of CNY 125,700 per QALY. The dashed line represents CNY 41,900 per QALY.

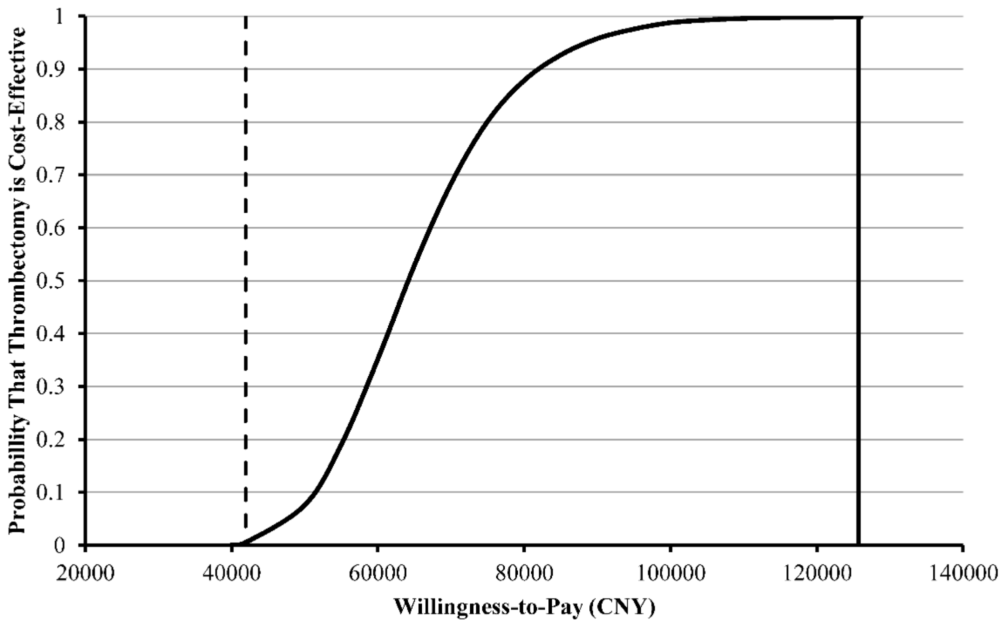

Supplement: Supplementary file 1 [file bmjopen-2017-018951supp001.pdf]
