# Supplementary material for: Cost-effectiveness of mechanical thrombectomy within 6 hours of acute ischaemic stroke in China
Source: BMJ Open. 2018 Feb 22;8(2):e018951. doi: 10.1136/bmjopen-2017-018951 (PMC5855394; doi:10.1136/bmjopen-2017-018951)
Supplement: Author's manuscript [file bmjopen-2017-018951.draft_revisions.pdf]

BMJ Open

BMJ Open is committed to open peer review. As part of this commitment we make the peer review history of every article we publish publicly available.

When an article is published we post the peer reviewers' comments and the authors' responses online. We also post the versions of the paper that were used during peer review. These are the versions that the peer review comments apply to.

The versions of the paper that follow are the versions that were submitted during the peer review process. They are not the versions of record or the final published versions. They should not be cited or distributed as the published version of this manuscript.

BMJ Open is an open access journal and the full, final, typeset and author-corrected version of record of the manuscript is available on our site with no access controls, subscription charges or pay-per-view fees (http://bmjopen.bmj.com).

If you have any questions on BMJ Open's open peer review process please email editorial.bmjopen@bmj.com

BMJ Open

Cost-Effectiveness of Mechanical Thrombectomy Within 6 Hours of Acute Ischaemic Stroke in China

Journal:	*BMJ Open*
Manuscript ID	bmjopen-2017-018951
Article Type:	Research
Date Submitted by the Author:	02-Aug-2017
Complete List of Authors:	PAN, YUESONG; Beijing Tiantan Hospital, Capital Medical University, Department of Neurology<br>Cai, Xueli; Lishui Hospital of Zhejiang University, Department of Neurology<br>Huo, Xiaochuan; Beijing Tiantan Hospital, Interventional Neurology;<br>Zhao, Xingquan; Beijing Tiantan Hospital, Neurology<br>Liu, Liping; Beijing Tiantan Hospital, Capital Medical University, Department of Neurology<br>Wang, Yongjun; Beijing Tiantan Hospital, Neurology<br>Miao, Zhongrong; Beijing Tiantan Hospital, Neurology<br>Wang, Yilong; Beijing Tiantan Hospital, Neurology
**Primary Subject Heading**:	Health economics
Secondary Subject Heading:	Neurology
Keywords:	cost-effectiveness, costs, quality-adjusted life-year, Stroke < NEUROLOGY, thrombectomy

SCHOLARONE™
Manuscripts

For peer review only

Cost-Effectiveness of Mechanical Thrombectomy Within 6 Hours of Acute Ischaemic Stroke in China

Cover title: CEA of mechanical thrombectomy of ischaemic stroke

Yuesong Pan,[1,2,3,4,5*] Xueli Cai,[6*] Xiaochuan Huo,[1,2,3,4] Xingquan Zhao,[1,2,3,4] Liping Liu,[1,2,3,4] Yongjun Wang,[1,2,3,4] Zhongrong Miao,[1,2,3,4] Yilong Wang[1,2,3,4]

[1] Department of Neurology, Beijing Tiantan Hospital, Capital Medical University, Beijing, China

[2] China National Clinical Research Center for Neurological Diseases, Beijing, China

[3] Center of Stroke, Beijing Institute for Brain Disorders, Beijing, China

[4] Beijing Key Laboratory of Translational Medicine for Cerebrovascular Disease, Beijing, China

[5] Department of Epidemiology and Health Statistics, School of Public Health, Capital Medical University, Beijing, China.

[6] Department of Neurology, Lishui Hospital of Zhejiang University (the Central Hospital of Lishui), Lishui, Zhejiang, China.

* Yuesong Pan and Xueli Cai contributed equally to the manuscript.

Corresponding author: Yilong Wang

Address: No.6 Tiantanxili, Dongcheng District, Beijing, China, 100050

Tel: 0086-010-67092222

Fax: 0086-010-67098542

E-mail: yilong528@gmail.com

Co-corresponding author: Zhongrong Miao **E-mail:** zhongrongm@163.com

Tables: 3; **Figure:** 3; **Supplemental table:** 1; **Supplemental figure:** 1; **Words:** 2732.

Key Words: cost-effectiveness; costs; quality-adjusted life-year; stroke; thrombectomy.

For peer review only

Abstract

Objectives Endovascular mechanical thrombectomy is an effective but expensive therapy for acute ischaemic stroke with proximal anterior circulation occlusion. This study aimed to determine the cost-effectiveness of mechanical thrombectomy in China, the largest developing country.

Design A combination of decision tree and Markov model was developed. Outcomes and costs data were derived from the published literature and claims database. Efficacy data were derived from the patient-level pooled analyses of the 5 trials in 2015. One-way and probabilistic sensitivity analyses were performed to assess the uncertainty of the results.

Setting Hospitals in China.

Participants Patients with acute ischaemic stroke caused by proximal anterior circulation occlusion within 6 hours.

Interventions Mechanical thrombectomy within 6 hours with IV tPA treatment within 4.5 hours versus IV tPA treatment alone.

Outcome measures Benefit was assessed by calculating the cost per quality-adjusted life-year (QALY) gained in the long term (30 years).

Results The addition of mechanical thrombectomy compared with standard treatment alone yielded a lifetime gain of 0.934 QALYs at an additional cost of CNY 61,760 (US$ 9,500), resulting in a cost of CNY 66,100 (US$ 10,170) per QALY gained. Probabilistic sensitivity analysis showed that mechanical thrombectomy is cost-effective in 91.9% of the simulation runs at a willingness-to-pay threshold of CNY 125,700 (US$ 19,300) per QALY.

Conclusions Mechanical thrombectomy for acute ischaemic stroke caused by proximal anterior circulation occlusion within 6 hours was cost-effective in China. This may be a reference to

For peer review only

medical resources allocation for stroke treatment in low- and middle-income countries and the remote areas in the developed countries.

For peer review only

Strengths and limitations of this study

- Strengths of this study include providing with information of cost-effectiveness analysis evaluating mechanical thrombectomy for ischaemic stroke in low- and middle-income countries.
- Results were robust across sensitivity and scenario analyses.
- A limitation of this study is that health status and costs as a result of causes other than stroke were not included in this model.
- An additional limitation is that the efficacy of mechanical thrombectomy treatment was based on trials completed in high income countries; however, this is the only data available.

For peer review only

Background

Arterial recanalization and subsequent reperfusion performed shortly after acute ischaemic stroke have demonstrated their ability to restore brain function.[1] Apart from intravenous recombinant tissue-type plasminogen activator (IV tPA) within 4.5 hours, endovascular mechanical thrombectomy is another effective reperfusion strategy, which can remove large, proximal clots rapidly and result in higher rate of reperfusion than IV tPA alone.[2] The recently published five clinical trials in 2015 have shown clear benefits of adding mechanical thrombectomy to standard care of acute ischaemic stroke caused by proximal anterior circulation occlusions.[2-6]

Although the effectiveness with acceptable safety of mechanical thrombectomy were well accepted, it is a costly therapy requiring expensive devices, highly trained proceduralists and special periprocedural support. Economic studies conducted in the USA, UK and Sweden showed mechanical thrombectomy treatment for acute ischaemic stroke is cost-effective or even cost-saving in the long term.[7-12] However, all of these studies were conducted in high income countries, which results may not be applied in low- and middle-income countries like China, where medical resources are scarce and stroke is a leading cause of death.[13] Little is known about the cost-effectiveness and feasibility of wide generalization of adding mechanical thrombectomy treatment to standard care in patients with acute ischaemic stroke in low- and middle-income countries. Economic analysis of mechanical thrombectomy in low- and middle-income countries is urgent. In this study, we model the cost-effectiveness of adding mechanical thrombectomy treatment to standard care for acute ischaemic stroke in the setting of the biggest low- and middle-income country-- China.

For peer review only

Methods

Model Overview

A combination of decision tree and Markov model (figure 1) was developed to simulate the short-term (1, 5, 6 years) and long-term (30 years) cost-effectiveness of mechanical thrombectomy using stent retrievers within 6 hours with IV tPA treatment within 4.5 hours versus IV tPA treatment alone within 4.5 hours after onset of stroke. The base case of model was a cohort of 100,000 patients with acute ischaemic stroke with proximal anterior circulation occlusion (36% female), with mean age of 63 years old, arriving at hospital within 6 hours after onset of stroke. The baseline characteristics were same as patients enrolled in the treated arm of the Endovascular therapy for Acute ischaemic Stroke Trial (EAST, a non-randomized interventional study to evaluate the safety and efficacy of Solitaire thrombectomy in Chinese patients with acute stroke),[14] who arrived at hospital within 6 hours. Among patients arrived at hospital within 6 hours after stroke onset, we assumed 85.4% patients arrived within 4.5 hours in both arms according to data from China National Stroke Registry (CNSR, a nationwide registry recruiting 21,902 consecutive patients with acute cerebrovascular events from 132 hospitals in China between September 2007 and August 2008.),[15] all of whom were assumed to receive IV tPA treatment; while all patients in the intervention arm received mechanical thrombectomy treatment. Total costs and quality-adjusted life-years (QALYs) gained with each alternative were estimated for each health state at 90 days from the index event and then estimated annually for the remainder 30 years. This analysis was conducted from the perspective of healthcare payers.

Input Parameters

Model input parameters were drawn from the published literature and the claims database (table 1; table 2). The effect and safety outcomes of mechanical thrombectomy-treated patients with

For peer review only - http://bmjopen.bmj.com/site/about/guidelines.xhtml

acute ischaemic stroke at day 90 were obtained from the recently published patient-level pooled analyses of the 5 trials, including the Multicenter Randomized Clinical Tiral of Endovascular Treatment for Acute Ischaemic Stroke in the Netherlands (MR CLEAN), the Endovascular Treatment for Small Core and Anterior Circulation Proximal Occlusion with Emphasis on Minimizing CT to Recanalization Times (ESCAPE), the Randomized Trial of Revascularization with Solitaire FR Device versus Best Medical Therapy in the Treatment of Acute Stroke Due to Anterior Circulation Large Vessel Occlusion Presenting within Eight Hours of Symptom Onset (REVASCAT), the Extending the Time for Thrombolysis in Emergency Neurological Deficits-Intra-Arterial (EXTEND IA) and the Solitaire with the Intention for Thrombectomy as Primary Endovascular Treatment (SWIFT PRIME) (table 1).[16]

Recurrent rates of stroke by modified Rankin Scale (mRS) categories and death rate with recurrent strokes in years after the first 90 days were estimated from the CNSR study.[15] We further assumed an increase in risk of stroke recurrence by 1.03-fold per life year.[17] Patients remaining alive after stroke recurrence were assumed to be reallocated equally among categories of equal and greater disability.[18] That is, patients in independent state (mRS 0-2) who had a recurrent stroke and survived were allocated equally among independent state (mRS 0-2) and dependent state (mRS 3-5); while patients in dependent state remaining alive after stroke recurrence were all remain in dependent state.

Age specific non-stroke death rates were derived from the most recent published census of China and adjusted by the causes of death of 2013 reported in the China Health Statistics Yearbook 2014.[19 20] Disability status were assumed to affect survival rate, therefore the final age specific non-stroke death rates for those with dependent state were adjusted by the hazard ratio of death for mRS 3-5.[21]

For peer review only

Costs

The total costs including both out-of-pocket costs and reimbursements, were converted to 2013 Chinese Yuan Renminbi (CNY) using the medical care component of consumer price index.[20] The average cost of one-time hospitalization and annual post-hospitalization costs (such as inpatient and outpatient rehabilitation and secondary preventive costs) after stroke according to categories of mRS were obtained from the database of the CNSR study.[15] The additional costs of mechanical thrombectomy, including costs for devices, procedure and special periprocedural care, were estimated using the data from the EAST study and the Thrombolysis Implementation and Monitor of acute ischaemic Stroke in China (TIMS-CHINA, a national prospective registry of 1,440 acute ischaemic stroke with thrombolytic therapy with IV tPA from 67 centers in China between May 2007 and April 2012.) study.[14 22] The additional costs of tPA treatment and occurrence of sICH were estimated using the data from CNSR and the TIMS-CHINA study. We did not include the indirect economic costs such as lost work productivity in this study. All costs and utilities were discounted by 3% per year.[23]

Health States

Patients could undergo transitions between three health states according to functional outcome based on mRS: independent (mRS 0-2), dependent (mRS 3-5) or dead (mRS 6).[7 24] At the end of each Markov cycle, patients could remain in their current health state, transit to a health state with greater disability due to recurrent stroke, or die due to a recurrent stroke or a non-stroke cause (figure 1).

Outcome Assessment

Health outcomes were measured in quality-adjusted life-years (QALYs) which were calculated by multiplying length of life by utility scores derived from the literature.[9 25 26] Utility scores of

For peer review only - http://bmjopen.bmj.com/site/about/guidelines.xhtml

different disability states after stroke were developed using European quality of life scale (EQ-5D) along with the Chinese preference weights in a Chinese stroke population.[26] In our model, events of recurrent stroke and sICH were considered temporary health states unless they resulted in death. All patients entering these health states were assumed to have a short-term disutility of 30 days for event of recurrent stroke and only 14 days for event of sICH.[9 25] Total economic costs were calculated by multiplying the number of patients in each state by the direct medical costs for that state. The incremental cost-effectiveness ratio (ICER) was calculated by dividing the difference of costs by the difference in QALYs between the two treatment alternatives. We modeled the costs and QALYs gained over the short-term (1, 5, 6 years) and the long-term (30 years). Mechanical thrombectomy was considered cost-effective if the ICER was less than CNY 125,700 (3x GDP per capita of China in 2013,[20] US$ 19,300) per QALY gained. This willingness-to-pay threshold was recommended by the Commission on Macroeconomics and Health of World Health Organization.[23]

Sensitivity Analysis

A deterministic one-way sensitivity analysis, varying the probabilities, utilities and costs, one at a time within the plausible ranges (table 2), was performed to evaluate the uncertainty of the long-term (30 years) results of the model. To determine how much worse mechanical thrombectomy could have performed but still produced a cost-effective ICER, we constructed two hypothetical worse outcomes for mechanical thrombectomy performance. This was accomplished by setting the odds ratios of mRS 0-2 at day 90 for mechanical thrombectomy treatment at its lower limits of 90% and 95% confidence intervals in the pooled analyses of the 5 trials,[16] representing unfavorable and worse unfavorable scenarios, respectively. For each

For peer review only

outcome scenario, we also constructed four hypothetical cost scenarios by setting 10% increase or 10%, 25% and 50% decrease of cost of mechanical thrombectomy.

Furthermore, a probabilistic sensitivity analysis was also undertaken to evaluate the stochastic uncertainty as all variables varied simultaneously. It was performed by using Monte Carlo simulation in Ersatz v1.3 (a bootstrap add-in for Microsoft Excel for Windows; EpiGear International Pty Ltd, Brisbane, Australia). We assumed costs followed a lognormal distribution and the probabilities and utilities followed a beta distribution. The simulation was run 10,000 times. The results were summarized using a scatter-plot and a cost-effectiveness acceptability curve.

Results

Base Case Analysis

Table 3 shows the costs, outcomes and ICER for the mechanical thrombectomy treatment calculated in the short term (1, 5, 6 years) and in the long term (30 years). In the base-case scenario, for a 63-year-old patient with acute ischaemic stroke caused by proximal anterior circulation occlusion within 6 hours after onset of stroke, mechanical thrombectomy would be cost-ineffective in the first five year, but become cost-effective from the sixth year onwards, using the threshold of CNY 125,700 (3x GDP per capita of China in 2013, US$ 19,300) as the willingness-to-pay per QALY. After 6 years, mechanical thrombectomy gained 0.524 QALYs at an additional cost of CNY 59,500 (US$ 9,150), yielding an ICER of CNY 113,550 (US$ 17,470) per QALY gained. In the long term (30 years), mechanical thrombectomy gained 0.934 QALYs at an additional cost of CNY 61,760 (US$ 9,500), yielding an ICER of CNY 66,100 (US$ 10,170) per QALY gained.

For peer review only

Sensitivity Analysis

The results of the deterministic one-way sensitivity analysis for the ICER of mechanical thrombectomy in the long term are showed in the tornado diagrams (figure 2). Overall, the ICER was most sensitive to the additional cost of mechanical thrombectomy, utility of independence (mRS 0-2) and odds ratio of favorable functional outcome (mRS 0-2) at day 90. Even if the additional cost of mechanical thrombectomy increased to CNY 91,094, the ICER of mechanical thrombectomy (CNY 98,533/QALY) was still within the threshold of the willingness-to-pay per QALY (CNY 125,700, 3x GDP per capita of China in 2013). In each hypothetical case scenario, mechanical thrombectomy continued to produce a benefit in QALYs (online supplementary table 1). Even in the worst hypothetical scenario with worse unfavorable effect outcome and an increase of 10% price of mechanical thrombectomy, mechanical thrombectomy continued to be cost-effective (ICER: CNY 96,920/QALY). In the hypothetical case scenario with base case effect outcome and a decrease of 50% price of mechanical thrombectomy, mechanical thrombectomy could be high cost-effective (ICER: CNY 33,845/QALY<CNY 41,900 [1x GDP per capita of China in 2013, US$ 6,400]/QALY).

Figure 3 shows the results of the probabilistic sensitivity analysis in the long term. Among the 10,000 simulation runs, mechanical thrombectomy was cost-effective in 91.9% of the simulations at a willingness-to-pay threshold of CNY 125,700 (3x GDP per capita of China in 2013, US$ 19,300) per QALY, and in 21.8% of the simulations at a threshold of CNY 41,900 (1x GDP per capita of China in 2013, US$ 6,400) per QALY. Online supplementary figure 1 shows the cost-effectiveness acceptability curve of mechanical thrombectomy.

For peer review only

Discussion

Our study indicated that mechanical thrombectomy for acute ischaemic stroke caused by proximal anterior circulation occlusion within 6 hours after onset of stroke was cost-effective from the sixth year onwards in China. A patient with acute ischaemic stroke treated with mechanical thrombectomy gain an ICER of CNY 66,100 per QALY in the long term, which was below 3x GDP per capita of China in 2013 (CNY 125,700). We also found that the ICER is more sensitive to additional cost of mechanical thrombectomy, utility of independence and odds ratio of favorable functional outcome at day 90.

Similar as the results of cost-effective completed in other high-income countries,[7-10] our study shows that mechanical thrombectomy treatment is also cost-effective in low- and middle-income countries like China. The lifetime gain of 0.934 QALYs for mechanical thrombectomy treatment for acute ischaemic stroke in our study was comparable to that evaluated in the high income countries (0.7 QALYs in the USA,[7] 1.05 QALYs in the UK[9] and 0.99 QALYs in Sweden[10]). The QALYs gain of mechanical thrombectomy treatment was relatively more than that of most of other treatments for stroke. For example, the lifetime QALYs gain was 0.42 for IV tPA treatment for acute ischaemic stroke within 4.5 hours,[27] and 0.17 for clopidogrel for secondary prevention of stroke comparing with aspirin.[28] This may be mainly because of the large magnitude of effect of mechanical thrombectomy (odds ratio 2.35).[16]

Mechanical thrombectomy treatment has been accepted as the standard of care for acute ischaemic stroke patients caused by proximal anterior circulation occlusions within 6 hours after symptom onset.[29 30] However, economic costs of mechanical thrombectomy treatment are extremely high, especially in low- and middle-income countries like China, where people suffer higher burden of stroke and intracranial atherosclerosis is much more prevalent than the Western

For peer review only

countries.[31] The additional costs of mechanical thrombectomy is approximately 5-fold of one-time hospitalization costs without tPA treatment and mechanical thrombectomy in China, comparing with only 1.5-fold in the USA.[7 8] Previous studies showed that there were socioeconomic disparities in the utilization of mechanical thrombectomy for acute ischaemic stroke and patients with low income or in remote areas had lower rates of mechanical thrombectomy utilization.[32 33] The implement of mechanical thrombectomy treatment are to-some-extent depend on the cost-effectiveness of the technology, which is of importance for the clinical decision in the low- and middle-income countries. Our study supports the implement of mechanical thrombectomy treatment after acute ischaemic stroke in clinical practice in the low- and middle-income countries from the perspective of economics, and may provide an important reference for the low income or remote areas in the Western countries.

The real-world implement of endovascular thrombectomy treatment in low- and middle-income countries and areas may be restricted by the poor awareness of the public, poor infrastructure, inefficient system and deficiency of specialists.[10] To generalize this new technology in these countries and areas, education targeted to the public, hospital administration and governmental agencies should be improved so that they can better understand the benefit and cost-effectiveness of thrombectomy.[34] Furthermore, service system redesign is needed to establish efficient care chains and workflow with coordination among neurointerventinalists and other departments. Additionally, high experience and skills are required to perform this advanced technology, while ways of providing interventional treatment are unstandardized and quite diverse in low- and middle- income countries like China.[34] Rapid efforts are urgently required to organize standardized training of performing thrombectomy to handle deficiency of intervention specialists in these countries and areas.

For peer review only

This study has several limitations that should be considered when interpreting the results. First, our model focused on the impact of mechanical thrombectomy treatment on acute ischaemic stroke, and health status and costs as a result of other causes, such as occurrence of intracranial hemorrhage and myocardial infarction, were not included in this model. Second, functional improvement after rehabilitation was not considered in the model for lack of data available on the efficacy of rehabilitation. However, organized rehabilitation after stroke in China is poor.[35 36] Third, we to some extent arbitrarily assumed that the patients with independent state remaining alive after stroke recurrence were equally reallocated in the categories of independent and dependent state. However, this is not unprecedented in the modeling of cost-effectiveness analysis for stroke.[18] Fourth, the efficacy of mechanical thrombectomy treatment was based on trials completed in high income countries. However, this is the only data available. These limitations would have led to under- or over- estimation of the true cost-effectiveness of mechanical thrombectomy treatment in low- and middle-income countries. However, the results were robust as shown in the sensitivity analyses.

Conclusions

Mechanical thrombectomy for acute ischaemic stroke caused by proximal anterior circulation occlusion within 6 hours after onset of stroke was cost-effective in China. Our study provides with constructive information on medical resources allocation for stroke treatment in low- and middle-income countries, and may also be a reference to the low income or remote areas in the developed countries.

For peer review only

Contributors

YSP, YJW and YLW designed the study and drafted the manuscript. YSP, XLC and XCH: collected the data, performed the literature search and constructed the decision tree. XQZ, LPL and ZRM interpreted the data and revised the manuscript.

Funding

This work was supported by grants from the Ministry of Science and Technology of the People's Republic of China (2015BAI12B04, 2015BAI12B02, 2016YFC0901000, 2016YFC0901001, 2016YFC0901002), grants from Beijing Municipal Science and Technology Commission (D151100002015001, D151100002015002, D151100002015003, Z15110200390000, Z151100003915117), and grants from Beijing Municipal Commission of Health and Family Planning (No.2016-1-2041, SML20150502).

Competing interests

None declared.

Data sharing statement

We, as the authors of this original research article, state that there are no additional, unpublished data available from this study.

For peer review only

References

1. Lees KR, Bluhmki E, von Kummer R, *et al*. Time to treatment with intravenous alteplase and outcome in stroke: an updated pooled analysis of ECASS, ATLANTIS, NINDS, and EPITHET trials. *Lancet* 2010;375:1695-703.
2. Berkhemer OA, Fransen PS, Beumer D, *et al*. A randomized trial of intraarterial treatment for acute ischemic stroke. *N Engl J Med* 2015;372:11-20.
3. Goyal M, Demchuk AM, Menon BK, *et al*. Randomized assessment of rapid endovascular treatment of ischemic stroke. *N Engl J Med* 2015;372:1019-30.
4. Saver JL, Goyal M, Bonafe A, *et al*. Stent-retriever thrombectomy after intravenous t-PA vs. t-PA alone in stroke. *N Engl J Med* 2015;372:2285-95.
5. Jovin TG, Chamorro A, Cobo E, *et al*. Thrombectomy within 8 hours after symptom onset in ischemic stroke. *N Engl J Med* 2015;372:2296-306.
6. Campbell BC, Mitchell PJ, Kleinig TJ, *et al*. Endovascular therapy for ischemic stroke with perfusion-imaging selection. *N Engl J Med* 2015;372:1009-18.
7. Leppert MH, Campbell JD, Simpson JR, Burke JF. Cost-Effectiveness of Intra-Arterial Treatment as an Adjunct to Intravenous Tissue-Type Plasminogen Activator for Acute Ischemic Stroke. *Stroke* 2015;46:1870-6.
8. Kunz WG, Hunink MG, Sommer WH, *et al*. Cost-Effectiveness of Endovascular Stroke Therapy: A Patient Subgroup Analysis From a US Healthcare Perspective. *Stroke* 2016;47:2797-804.
9. Ganesalingam J, Pizzo E, Morris S, Sunderland T, Ames D, Lobotesis K. Cost-Utility Analysis of Mechanical Thrombectomy Using Stent Retrievers in Acute Ischemic Stroke. *Stroke* 2015;46:2591-8.

For peer review only

10. Aronsson M, Persson J, Blomstrand C, Wester P, Levin LÅ. Cost-effectiveness of endovascular thrombectomy in patients with acute ischemic stroke. *Neurology* 2016;86:1053-9.

11. Lobotesis K, Veltkamp R, Carpenter IH, Claxton LM, Saver JL, Hodgson R. Cost-effectiveness of Stent-retriever Thrombectomy in combination with IV t-PA Compared with IV t-PA Alone for Acute Ischemic Stroke in the UK. *J Med Econ* 2016:1-37.

12. Steen Carlsson K, Andsberg G, Petersson J, Norrving B. Long-term cost-effectiveness of thrombectomy for acute ischaemic stroke in real life: An analysis based on data from the Swedish Stroke Register (Riksstroke). *Int J Stroke* 2017:1747493017701154.

13. Yang G, Wang Y, Zeng Y, *et al*. Rapid health transition in China, 1990-2010: findings from the Global Burden of Disease Study 2010. *Lancet* 2013;381:1987-2015.

14. Miao Z, Huo X, Gao F, *et al*. Endovascular therapy for Acute ischemic Stroke Trial (EAST): study protocol for a prospective, multicentre control trial in China. *Stroke Vasc Neurol* 2016;1:44-51.

15. Wang Y, Cui L, Ji X, *et al*. The China National Stroke Registry for patients with acute cerebrovascular events: design, rationale, and baseline patient characteristics. *Int J Stroke* 2011;6:355-61.

16. Goyal M, Menon BK, van Zwam WH, *et al*. Endovascular thrombectomy after large-vessel ischaemic stroke: a meta-analysis of individual patient data from five randomised trials. *Lancet* 2016;387:1723-31.

17. Pennlert J, Eriksson M, Carlberg B, Wiklund PG. Long-term risk and predictors of recurrent stroke beyond the acute phase. *Stroke* 2014;45:1839-41.

For peer review only

18. Tung CE, Win SS, Lansberg MG. Cost-effectiveness of tissue-type plasminogen activator in the 3- to 4.5-hour time window for acute ischemic stroke. *Stroke* 2011;42:2257-62.

19. National Bureau of Statistics of China. The 2010 population census of the peaple's republic of china. Websit: http://www.stats.gov.cn/tjsj/pcsj/rkpc/6rp/indexch.htm (in Chinese). [Accessed Feb 25, 2016].

20. Ministry of Health of the People's Republic of China. China Health Statistics Yearbook 2014. Peking Union Medical College Press. Beijing (2014) (in Chinese).

21. Samsa GP, Reutter RA, Parmigiani G, *et al*. Performing cost-effectiveness analysis by integrating randomized trial data with a comprehensive decision model: application to treatment of acute ischemic stroke. *J Clin Epidemiol* 1999;52:259-71.

22. Liao XL, Wang CX, Wang YL, *et al*. Implementation and outcome of thrombolysis with alteplase 3 to 4.5 h after acute stroke in Chinese patients. *CNS Neurosci Ther* 2013;19:43-7.

23. Pan Y, Wang A, Liu G, *et al*. Cost-effectiveness of clopidogrel-aspirin versus aspirin alone for acute transient ischemic attack and minor stroke. *J Am Heart Assoc* 2014;3:e000912.

24. Kim AS, Nguyen-Huynh M, Johnston SC. A cost-utility analysis of mechanical thrombectomy as an adjunct to intravenous tissue-type plasminogen activator for acute large-vessel ischemic stroke. *Stroke* 2011;42:2013-8.

25. Lee S, Anglade MW, Pham D, Pisacane R, Kluger J, Coleman CI. Cost-effectiveness of rivaroxaban compared to warfarin for stroke prevention in atrial fibrillation. *Am J Cardiol* 2012;110:845-51.

26. Wang YL, Pan YS, Zhao XQ, *et al*. Recurrent stroke was associated with poor quality of life in patients with transient ischemic attack or minor stroke: finding from the CHANCE trial. *CNS Neurosci Ther* 2014;20:1029-35.

For peer review only

27. Pan Y, Chen Q, Zhao X, *et al*. Cost-Effectiveness of Thrombolysis within 4.5 Hours of Acute Ischemic Stroke in China. *PLoS One* 2014;9:e110525.

28. Schleinitz MD, Weiss JP, Owens DK. Clopidogrel versus aspirin for secondary prophylaxis of vascular events: a cost-effectiveness analysis. *Am J Med* 2004;116:797-806.

29. Powers WJ, Derdeyn CP, Biller J, *et al*. 2015 American Heart Association/American Stroke Association Focused Update of the 2013 Guidelines for the Early Management of Patients With Acute Ischemic Stroke Regarding Endovascular Treatment: A Guideline for Healthcare Professionals From the American Heart Association/American Stroke Association. *Stroke* 2015;46:3020-35.

30. Miao Z, Wang Y. Chinese guidelines for endovascular treatment for acute ischemic stroke 2015.(in Chinese). *Chin J Stroke* 2015;10:590-606.

31. Wang Y, Zhao X, Liu L, *et al*. Prevalence and outcomes of symptomatic intracranial large artery stenoses and occlusions in China: the Chinese Intracranial Atherosclerosis (CICAS) Study. *Stroke* 2014;45:663-9.

32. Brinjikji W, Rabinstein AA, Cloft HJ. Socioeconomic disparities in the utilization of mechanical thrombectomy for acute ischemic stroke. *J Stroke Cerebrovasc Dis* 2014;23:979-84.

33. Pérez de la Ossa N, Abilleira S, Dorado L, *et al*. Access to Endovascular Treatment in Remote Areas: Analysis of the Reperfusion Treatment Registry of Catalonia. *Stroke* 2016;47:1381-4.

34. Miao Z. New era of stroke therapy and new challenges. *Stroke Vasc Neurol* 2016;1:6-7.

For peer review only

35. Li AD, Huang ZQ, Liu HT, Deng YT, Xiao JW, Liang JW. Knowledge, Attitude and Behavior for Stroke and Rehabilitation in Stroke Patients and Relatives.(in Chinese). *Chin J Rehabil Theory Pract* 2009;15:252-4.

36. Gao YL, Chen LD, Tao J, Chen SM. Investigation on the investment and popularization of rehabilitation from cerebral apoplexy: in 2 communities of Xiamen City.(in Chinese). *Chin J Convalescent Med* 2009;18:472-4.

For peer review only

Figure Legends

Figure 1. Decision tree and Markov state transition model. A patient with an acute ischemic stroke with anterior circulation occlusion entered the model at 63 years old receiving either intravenous tissue-type plasminogen activator (IV tPA) with or without mechanical thrombectomy, and transited between health states until death or 30 years. Patients may remain in the same health state, move to a state of equal or greater disability after recurrent stroke, or die. Only transition from dependent state (modified Rankin Scale [mRS] 3-5) was illustrated in the figure. M indicates Markov node; sICH, symptomatic intracerebral hemorrhage.

Figure 2. One-way sensitivity analysis on incremental cost-effectiveness ratio (ICER) gained in the long term (30 years) by mechanical thrombectomy. All model input parameters were analyzed, and only ten parameters with the highest relative effects on ICER are displayed. Base-case scenario of ICER is CNY 66,124 per quality-adjusted life-year (QALY) gained.

Figure 3. Scatterplot of the result of probabilistic sensitivity analysis in the long term (30 years). Each point represents a simulation. The dark square represents the base case (0.934 QALYs gained at an incremental cost of CNY 61,760). The solid line represents the willingness-to-pay threshold of CNY 125,700 per QALY. The dashed line represents CNY 41,900 per QALY. Points to the right of the solid line are considered cost-effective.

For peer review only

Tables

Table 1. Efficacy and Safety Outcome of Mechanical Thrombectomy for Acute Ischemic Stroke

Percentage of outcomes at 90 days	Mechanical thrombectomy + IV tPA	IV tPA	Reference
mRS 0-2	46.0	26.5	[16]
mRS 3-5	38.7	54.6	
Death (mRS 6)	15.3	18.9	
sICH	4.4	4.3	

IV tPA, intravenous tissue plasminogen activator; mRS, modified Rankin Scale; sICH, symptomatic intracerebral hemorrhage.

For peer review only

Table 2. Base-case and Plausible Ranges of Model Inputs

Model Input	Base Case	Range	Reference
**Probabilities inputs**			
Proportion of patients arrived within 4.5 hours	0.854		CNSR
Recurrent rate of stroke (per patient year)			CNSR
mRS 0-2	0.1026	0.0961-0.1093	
mRS 3-5	0.1418	0.1303-0.1534	
Relative risk of stroke recurrence per life year	1.03	1.02-1.04	[17]
Death with recurrent stroke	0.2101	0.1887-0.2316	CNSR
Age specific non-stroke death rate *	0.0089-0.1653		[19 20]
Hazard ratio of non-stroke death for mRS 3-5	1.78	1.43-2.14	[21]
**Cost inputs (2013 Chinese Yuan Renminbi)**			
Additional costs of mechanical thrombectomy	60821	48525-91094	EAST
Additional costs of tPA treatment	11179	8655-13037	CNSR, TIMS-CHINA
Additional costs of sICH	2374	516-4955	TIMS-CHINA
One-time hospitalization costs			CNSR
mRS 0-2	10055	5774-12780	
mRS 3-5	13729	7604-18438	
mRS 6	11121	5225-14699	
Annual post-hospitalization costs			CNSR
mRS 0-2	7385	2003-8900	
mRS 3-5	11350	3376-15487	

**Utility inputs**			
mRS 0-2	0.76	0.60-0.96	[26]
mRS 3-5	0.21	0.11-0.29	[26]
Death (mRS 6)	0	0.00-0.00	[26]
Recurrent stroke	0.34	0.32-0.36	[9]
sICH	0.84	0.7-1.0	[25]
**Discount rate inputs**			
Costs	0.03	0.03-0.08	[23]
Outcomes	0.03	±20%	[23]

All costs were converted to 2013 Chinese Yuan Renminbi by using the medical care component of consumer price index; to convert CNY to US dollars, divide by 6.5.

IV tPA, intravenous tissue plasminogen activator; mRS, modified Rankin Scale; sICH, symptomatic intracerebral hemorrhage; CNSR, China National Stroke Registry; EAST, Endovascular therapy for Acute ischemic Stroke Trial; TIMS-CHINA, Thrombolysis Implementation and Monitor of acute ischemic Stroke in China.

* Age specific non-stroke death rate: only the number of 63 years old (0.0089) and 93 years old (0.1653) are presented.

For peer review only

Table 3. Costs and Outcomes in Base-case Analysis

Time horizon	Treat strategy	QALYs	Cost (CNY)	ICER (CNY/QALY)
1 year	IV tPA alone	0.373	30,810	-
	Mechanical thrombectomy + IV tPA	0.495	91,210	495,082
5 years	IV tPA alone	1.307	62,260	-
	Mechanical thrombectomy + IV tPA	1.766	121,820	129,760
6 years	IV tPA alone	1.488	68,810	-
	Mechanical thrombectomy + IV tPA	2.012	128,310	113,550
30 years	IV tPA alone	2.684	119,620	-
	Mechanical thrombectomy + IV tPA	3.618	181,380	66,124

IV tPA, intravenous tissue plasminogen activator; QALY, quality-adjusted life-year; ICER, incremental cost-effectiveness ratio.

review only

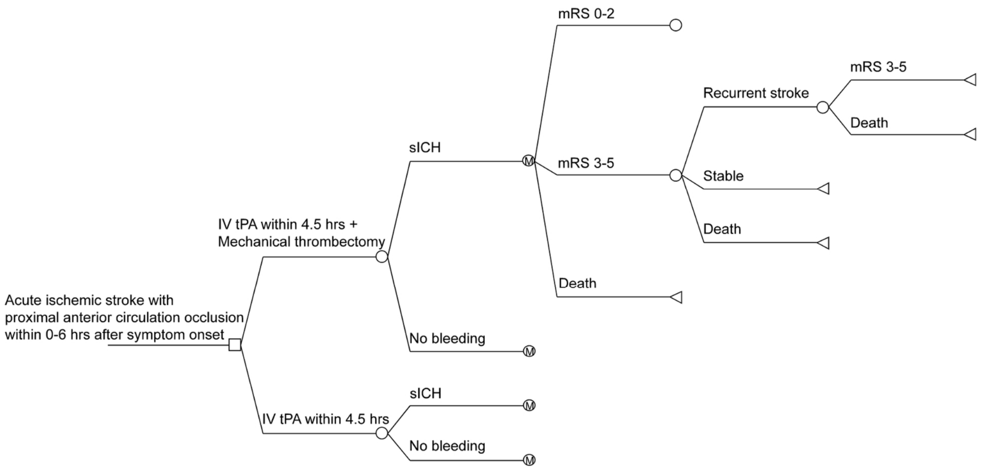

Figure 1. Decision tree and Markov state transition model. A patient with an acute ischemic stroke with anterior circulation occlusion entered the model at 63 years old receiving either intravenous tissue-type plasminogen activator (IV tPA) with or without mechanical thrombectomy, and transited between health states until death or 30 years. Patients may remain in the same health state, move to a state of equal or greater disability after recurrent stroke, or die. Only transition from dependent state (modified Rankin Scale [mRS] 3-5) was illustrated in the figure. M indicates Markov node; sICH, symptomatic intracerebral hemorrhage.

83x40mm (600 x 600 DPI)

view only

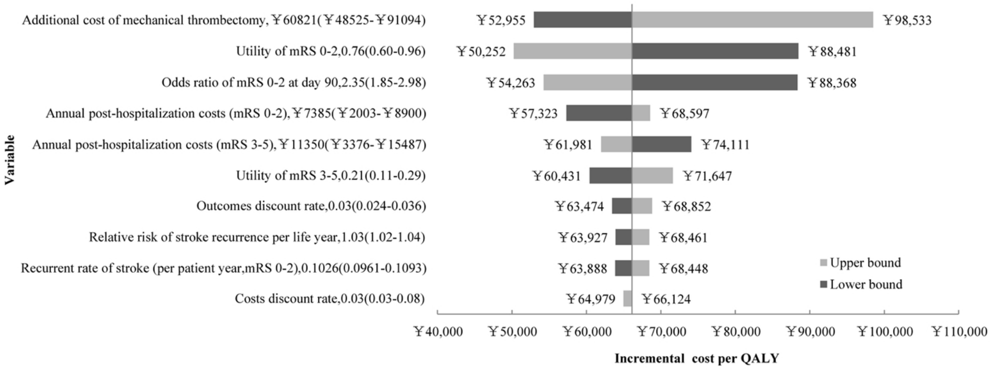

Figure 2. One-way sensitivity analysis on incremental cost-effectiveness ratio (ICER) gained in the long term (30 years) by mechanical thrombectomy. All model input parameters were analyzed, and only ten parameters with the highest relative effects on ICER are displayed. Base-case scenario of ICER is CNY 66,124 per quality-adjusted life-year (QALY) gained.

64x24mm (600 x 600 DPI)

For peer review only

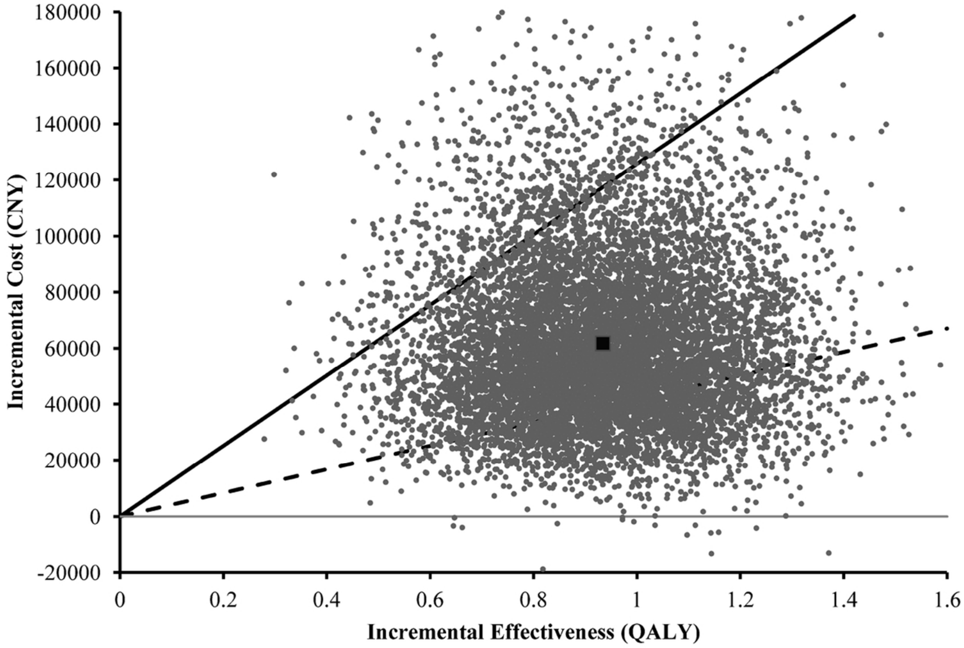

Figure 3. Scatterplot of the result of probabilistic sensitivity analysis in the long term (30 years). Each point represents a simulation. The dark square represents the base case (0.934 QALYs gained at an incremental cost of CNY 61,760). The solid line represents the willingness-to-pay threshold of CNY 125,700 per QALY. The dashed line represents CNY 41,900 per QALY. Points to the right of the solid line are considered cost-effective.

55x37mm (600 x 600 DPI)

ONLINE SUPPLEMENTARY MATERIALS

Table 1. Hypothetical Worse Performance and Costs of Mechanical Thrombectomy, ICER (CNY/QALY)

Additional cost of mechanical thrombectomy	Outcome of mechanical thrombectomy: Base Case (OR=2.35)*	Unfavorable (OR=1.93)	Worse unfavorable (OR=1.85)
+10%, CNY 66903	73,040	91,423	96,920
Base case, CNY 60821	66,124	83,338	88,368
-10%, CNY 54739	59,979	75,253	79,817
-25%, CNY 45616	50,172	63,112	66,976
-50%, CNY 30410	33,845	42,899	45,598

ICER, incremental cost-effectiveness ratio; QALY, quality-adjusted life-year; OR, Odds ratio.

* OR: OR of modified Rankin Scale 0-2 at day 90 for mechanical thrombectomy.

For peer review only

Figure 1. Cost-effectiveness acceptability curve. The curve presents the probability that mechanical thrombectomy within 6 hours to be cost-effective against willingness-to-pay threshold. The solid line represents the willingness-to-pay threshold of CNY 125,700 per QALY. The dashed line represents CNY 41,900 per QALY.

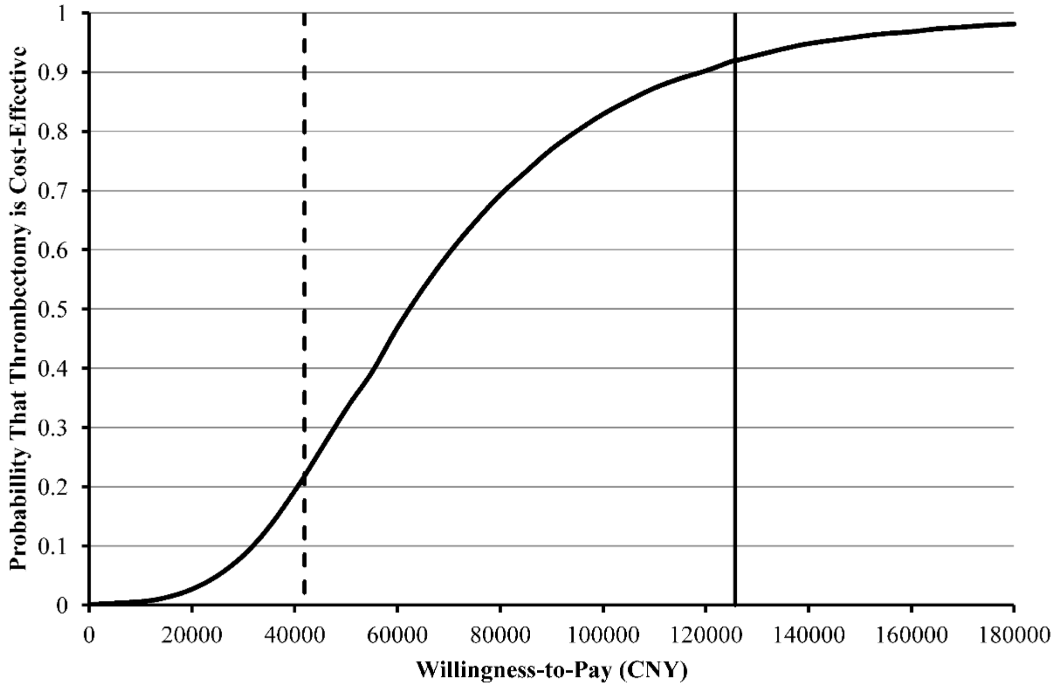

For peer review only

BMJ 2013;346:f1049 doi: 10.1136/bmj.f1049 (Published 25 March 2013)

Table

Table 1| **CHEERS checklist—Items to include when reporting economic evaluations of health interventions**

Section/item	Item No	Recommendation	Reported on page No/ line No
**Title and abstract**			
Title	1	Identify the study as an economic evaluation or use more specific terms such as "cost-effectiveness analysis", and describe the interventions compared.	Title Page
Abstract	2	Provide a structured summary of objectives, perspective, setting, methods (including study design and inputs), results (including base case and uncertainty analyses), and conclusions.	Page 1
**Introduction**			
Background and objectives	3	Provide an explicit statement of the broader context for the study.	Page 4
		Present the study question and its relevance for health policy or practice decisions.	Page 4
**Methods**			
Target population and subgroups	4	Describe characteristics of the base case population and subgroups analysed, including why they were chosen.	Page 5
Setting and location	5	State relevant aspects of the system(s) in which the decision(s) need(s) to be made.	Page 5
Study perspective	6	Describe the perspective of the study and relate this to the costs being evaluated.	Page 5
Comparators	7	Describe the interventions or strategies being compared and state why they were chosen.	Page 5
Time horizon	8	State the time horizon(s) over which costs and consequences are being evaluated and say why appropriate.	Page 5
Discount rate	9	Report the choice of discount rate(s) used for costs and outcomes and say why appropriate.	Page 7
Choice of health outcomes	10	Describe what outcomes were used as the measure(s) of benefit in the evaluation and their relevance for the type of analysis performed.	Page 7-8
Measurement of effectiveness	11a	*Single study-based estimates:* Describe fully the design features of the single effectiveness study and why the single study was a sufficient source of clinical effectiveness data.	
	11b	*Synthesis-based estimates:* Describe fully the methods used for identification of included studies and synthesis of clinical effectiveness data.	Page 6
Measurement and valuation of preference based outcomes	12	If applicable, describe the population and methods used to elicit preferences for outcomes.	Page 7-8
Estimating resources and costs	13a	*Single study-based economic evaluation:* Describe approaches used to estimate resource use associated with the alternative interventions. Describe primary or secondary research methods for valuing each resource item in terms of its unit cost. Describe any adjustments made to approximate to opportunity costs.	Page 7
	13b	*Model-based economic evaluation:* Describe approaches and data sources used to estimate resource use associated with model health states. Describe primary or secondary research methods for valuing each resource item in terms of its unit cost. Describe any adjustments made to approximate to opportunity costs.	
Currency, price date, and conversion	14	Report the dates of the estimated resource quantities and unit costs. Describe methods for adjusting estimated unit costs to the year of reported costs if necessary. Describe methods for converting costs into a common currency base and the exchange rate.	Page 7
Choice of model	15	Describe and give reasons for the specific type of decision-analytical model used. Providing a figure to show model structure is strongly recommended.	Page 5
Assumptions	16	Describe all structural or other assumptions underpinning the decision-analytical model.	Page 5
Analytical methods	17	Describe all analytical methods supporting the evaluation. This could include methods for dealing with skewed, missing, or censored data; extrapolation methods; methods for pooling data; approaches to validate or make adjustments (such as half cycle corrections) to a model; and methods for handling population heterogeneity and uncertainty.	Page 8-9
**Results**			
Study parameters	18	Report the values, ranges, references, and, if used, probability distributions for all parameters. Report reasons or sources for distributions used to represent uncertainty where appropriate. Providing a table to show the input values is strongly recommended.	Table 1,2
Incremental costs and outcomes	19	For each intervention, report mean values for the main categories of estimated costs and outcomes of interest, as well as mean differences between the comparator groups. If applicable, report incremental cost-effectiveness ratios.	Page 9,Table 3
Characterising uncertainty	20a	*Single study-based economic evaluation:* Describe the effects of sampling uncertainty for the estimated incremental cost and incremental effectiveness parameters, together with the impact of methodological assumptions (such as discount rate, study perspective).	Page 10

For peer review only

No commercial reuse: See rights and reprints http://www.bmj.com/permissions Subscribe: http://www.bmj.com/subscribe

(continued)

Section/item	Item No	Recommendation	Reported on page No/ line No
	20b	*Model-based economic evaluation:* Describe the effects on the results of uncertainty for all input parameters, and uncertainty related to the structure of the model and assumptions.	Page 10
Characterising heterogeneity	21	If applicable, report differences in costs, outcomes, or cost-effectiveness that can be explained by variations between subgroups of patients with different baseline characteristics or other observed variability in effects that are not reducible by more information.	Page 10
**Discussion**			
Study findings, limitations, generalisability, and current knowledge	22	Summarise key study findings and describe how they support the conclusions reached. Discuss limitations and the generalisability of the findings and how the findings fit with current knowledge.	Page 11-12
**Other**			
Source of funding	23	Describe how the study was funded and the role of the funder in the identification, design, conduct, and reporting of the analysis. Describe other non-monetary sources of support.	Page 14
Conflicts of interest	24	Describe any potential for conflict of interest of study contributors in accordance with journal policy. In the absence of a journal policy, we recommend authors comply with International Committee of Medical Journal Editors recommendations.	Page 14

For consistency, the CHEERS statement checklist format is based on the format of the CONSORT statement checklist

For peer review only

No commercial reuse: See rights and reprints http://www.bmj.com/permissions Subscribe: http://www.bmj.com/subscribe

BMJ Open

Cost-Effectiveness of Mechanical Thrombectomy within 6 Hours of Acute Ischaemic Stroke in China

Journal:	*BMJ Open*
Manuscript ID	bmjopen-2017-018951.R1
Article Type:	Research
Date Submitted by the Author:	27-Nov-2017
Complete List of Authors:	PAN, YUESONG; Beijing Tiantan Hospital, Capital Medical University, Department of Neurology<br>Cai, Xueli; Lishui Hospital of Zhejiang University, Department of Neurology<br>Huo, Xiaochuan; Beijing Tiantan Hospital, Interventional Neurology;<br>Zhao, Xingquan; Beijing Tiantan Hospital, Neurology<br>Liu, Liping; Beijing Tiantan Hospital, Capital Medical University, Department of Neurology<br>Wang, Yongjun; Beijing Tiantan Hospital, Neurology<br>Miao, Zhongrong; Beijing Tiantan Hospital, Neurology<br>Wang, Yilong; Beijing Tiantan Hospital, Neurology
**Primary Subject Heading**:	Health economics
Secondary Subject Heading:	Neurology
Keywords:	cost-effectiveness, costs, quality-adjusted life-year, Stroke < NEUROLOGY, thrombectomy

SCHOLARONE™
Manuscripts

For peer review only - http://bmjopen.bmj.com/site/about/guidelines.xhtml

Cost-Effectiveness of Mechanical Thrombectomy within 6 Hours of Acute Ischaemic Stroke in China

Cover title: CEA of mechanical thrombectomy of ischaemic stroke

Yuesong Pan,[1,2,3,4,5*] Xueli Cai,[6*] Xiaochuan Huo,[1,2,3,4] Xingquan Zhao,[1,2,3,4] Liping Liu,[1,2,3,4] Yongjun Wang,[1,2,3,4] Zhongrong Miao,[1,2,3,4] Yilong Wang[1,2,3,4]

[1] Department of Neurology, Beijing Tiantan Hospital, Capital Medical University, Beijing, China

[2] China National Clinical Research Centre for Neurological Diseases, Beijing, China

[3] Centre of Stroke, Beijing Institute for Brain Disorders, Beijing, China

[4] Beijing Key Laboratory of Translational Medicine for Cerebrovascular Disease, Beijing, China

[5] Department of Epidemiology and Health Statistics, School of Public Health, Capital Medical University, Beijing, China.

[6] Department of Neurology, Lishui Hospital of Zhejiang University (the Central Hospital of Lishui), Lishui, Zhejiang, China.

* Yuesong Pan and Xueli Cai contributed equally to the manuscript.

Corresponding author: Yilong Wang

Address: No.6 Tiantanxili, Dongcheng District, Beijing, China, 100050

Tel: 0086-010-67092222

Fax: 0086-010-67098542

E-mail: yilong528@gmail.com

Co-corresponding author: Zhongrong Miao **E-mail:** zhongrongm@163.com

Tables: 2; **Figure:** 3; **Supplemental table:** 1; **Supplemental figure:** 1; **Words:** 2940.

Key Words: cost-effectiveness; costs; quality-adjusted life-year; stroke; thrombectomy.

For peer review only

Abstract

Objectives Endovascular mechanical thrombectomy is an effective but expensive therapy for acute ischaemic stroke with proximal anterior circulation occlusion. This study aimed to determine the cost-effectiveness of mechanical thrombectomy in China, the largest developing country.

Design A combination of decision tree and Markov model was developed. Outcomes and costs data were derived from the published literature and claims database. Efficacy data were derived from the patient-level pooled analyses of the 5 trials in 2015. One-way and probabilistic sensitivity analyses were performed to assess the uncertainty of the results.

Setting Hospitals in China.

Participants Patients with acute ischaemic stroke caused by proximal anterior circulation occlusion within 6 hours.

Interventions Mechanical thrombectomy within 6 hours with IV tPA treatment within 4.5 hours versus IV tPA treatment alone.

Outcome measures Benefit was assessed by calculating the cost per quality-adjusted life-year (QALY) gained in the long term (30 years).

Results The addition of mechanical thrombectomy compared with standard treatment alone yielded a lifetime gain of 0.930 QALYs at an additional cost of CNY 54,600 (US$ 8,400), resulting in a cost of CNY 58,700 (US$ 9,030) per QALY gained. Probabilistic sensitivity analysis showed that mechanical thrombectomy is cost-effective in 95.4% of the simulation runs at a willingness-to-pay threshold of CNY 125,700 (US$ 19,300) per QALY.

Conclusions Mechanical thrombectomy for acute ischaemic stroke caused by proximal anterior circulation occlusion within 6 hours was cost-effective in China. This may be a reference to

For peer review only

medical resources allocation for stroke treatment in low- and middle-income countries and the remote areas in the developed countries.

For peer review only

Strengths and limitations of this study

- A combination of decision tree and Markov model was developed to simulate the short-term and long-term costs and outcomes after mechanical thrombectomy for ischaemic stroke.
- Most parameters used in the model, including costs, utilities and transition probabilities, were collected based on the Chinese setting, reflecting the situation in the low- and middle-income countries.
- A limitation of this study is that health status and costs as a result of causes other than stroke were not included in this model.
- An additional limitation is that the efficacy of mechanical thrombectomy treatment was based on trials completed in high income countries; however, this is the only data available.

For peer review only

Background

Arterial recanalisation and subsequent reperfusion performed shortly after acute ischaemic stroke have demonstrated their ability to restore brain function.[1] Apart from intravenous recombinant tissue-type plasminogen activator (IV tPA) within 4.5 hours, endovascular mechanical thrombectomy is another effective reperfusion strategy, which can remove large, proximal clots rapidly and result in higher rate of reperfusion than IV tPA alone.[2] Second-generation retrievable stents achieved higher recanalisation rates compared with first-generation devices. Although trials used first-generation thrombectomy devices failed to demonstrate clinical benefit compared with IV tPA, the recently published five clinical trials in 2015 with second-generation devices have shown clear benefits of adding mechanical thrombectomy to standard care of acute ischaemic stroke caused by proximal anterior circulation occlusions.[2-6]

Although the effectiveness with acceptable safety of mechanical thrombectomy with second generation devices were well accepted, it is a costly therapy requiring expensive devices, highly trained proceduralists and special periprocedural support. Economic studies conducted in the USA, UK, Sweden, Canada and Spain showed mechanical thrombectomy treatment for acute ischaemic stroke is cost-effective or even cost-saving in the long term.[7-15] However, all of these studies were conducted in high income countries, which results may not be applied in low- and middle-income countries like China, where medical resources are scarce and stroke is a leading cause of death.[16] Little is known about the cost-effectiveness and feasibility of wide generalization of adding mechanical thrombectomy treatment to standard care in patients with acute ischaemic stroke in low- and middle-income countries. Economic analysis of mechanical thrombectomy in low- and middle-income countries is urgent. In this study, we aimed to evaluate the cost-effectiveness of adding mechanical thrombectomy treatment with second generation

For peer review only

devices to standard care for acute ischaemic stroke in the setting of the biggest low- and middle-income country-- China.

Methods

Model Overview

A combination of decision tree and Markov model (figure 1) was developed to simulate the short-term (1, 4, 5 years) and long-term (30 years) cost-effectiveness of mechanical thrombectomy using stent retrievers within 6 hours with IV tPA treatment within 4.5 hours versus IV tPA treatment alone within 4.5 hours after onset of stroke. The base case of model was a cohort of 100,000 patients with acute ischaemic stroke with proximal anterior circulation occlusion (36% female), with mean age of 63 years old, arriving at hospital within 6 hours after onset of stroke. The baseline characteristics were same as patients enrolled in the treated arm of the Endovascular therapy for Acute ischaemic Stroke Trial (EAST, a non-randomized interventional study to evaluate the safety and efficacy of Solitaire thrombectomy in Chinese patients with acute stroke),[17] who arrived at hospital within 6 hours. Among patients arrived at hospital within 6 hours after stroke onset, we assumed 85.4% patients arrived within 4.5 hours in both arms according to data from China National Stroke Registry (CNSR, a nationwide registry recruiting 21,902 consecutive patients with acute cerebrovascular events from 132 hospitals in China between September 2007 and August 2008.),[18] all of whom were assumed to receive IV tPA treatment; while all patients in the intervention arm received mechanical thrombectomy treatment. Total costs and quality-adjusted life-years (QALYs) gained with each alternative were estimated for each health state at 90 days from the index event and then estimated annually for the remainder 30 years. This analysis was conducted from the perspective of healthcare payers.

For peer review only

This study did not involve human subjects and therefore was exempt from institutional review board approval.

Input Parameters

Model input parameters were drawn from the published literature and the claims database (table 1). The effect and safety outcomes of mechanical thrombectomy-treated patients with acute ischaemic stroke at day 90 were obtained from the recently published patient-level pooled analyses of the 5 trials, including the Multicentre Randomized Clinical Trial of Endovascular Treatment for Acute Ischaemic Stroke in the Netherlands (MR CLEAN), the Endovascular Treatment for Small Core and Anterior Circulation Proximal Occlusion with Emphasis on Minimizing CT to Recanalisation Times (ESCAPE), the Randomized Trial of Revascularization with Solitaire FR Device versus Best Medical Therapy in the Treatment of Acute Stroke Due to Anterior Circulation Large Vessel Occlusion Presenting within Eight Hours of Symptom Onset (REVASCAT), the Extending the Time for Thrombolysis in Emergency Neurological Deficits-Intra-Arterial (EXTEND IA) and the Solitaire with the Intention for Thrombectomy as Primary Endovascular Treatment (SWIFT PRIME) (table 1).[19]

Recurrent rates of stroke by modified Rankin Scale (mRS) categories and death rate with recurrent strokes in years after the first 90 days were estimated from the CNSR study.[18] We further assumed an increase in risk of stroke recurrence by 1.03-fold per life year.[20] Patients remaining alive after stroke recurrence were assumed to be reallocated equally among categories of equal and greater disability.[21] That is, patients in independent state (mRS 0-2) who had a recurrent stroke and survived were allocated equally among independent state (mRS 0-2) and dependent state (mRS 3-5); while patients in dependent state remaining alive after stroke recurrence were all remain in dependent state.

For peer review only

Age specific non-stroke death rates were derived from the most recent published census of China and adjusted by the causes of death of 2013 reported in the China Health Statistics Yearbook 2014.[22 23] Disability status were assumed to affect survival rate, therefore the final age specific non-stroke death rates for those with dependent state were adjusted by the hazard ratio of death for mRS 3-5.[24]

Costs

The total costs including both out-of-pocket costs and reimbursements, were converted to 2013 Chinese Yuan Renminbi (CNY) using the medical care component of consumer price index.[23] The average cost of one-time hospitalization and annual post-hospitalization costs (such as inpatient and outpatient rehabilitation and secondary preventive costs) after stroke according to categories of mRS were obtained from the database of the CNSR study.[18] The additional costs of mechanical thrombectomy, including costs for devices, procedure and special periprocedural care, were estimated using the data from the EAST study and the Thrombolysis Implementation and Monitor of acute ischaemic Stroke in China (TIMS-CHINA, a national prospective registry of 1,440 acute ischaemic stroke with thrombolytic therapy with IV tPA from 67 centres in China between May 2007 and April 2012.) study.[17 25] The additional costs of tPA treatment and occurrence of sICH were estimated using the data from CNSR and the TIMS-CHINA study. We did not include the indirect economic costs such as lost work productivity in this study. All costs and utilities were discounted by 3% per year.[26]

Health States

Patients could undergo transitions between three health states according to functional outcome based on mRS: independent (mRS 0-2), dependent (mRS 3-5) or dead (mRS 6).[7 27] At the end of each Markov cycle, patients could remain in their current health state, transit to a health state

For peer review only - http://bmjopen.bmj.com/site/about/guidelines.xhtml

with greater disability due to recurrent stroke, or die due to a recurrent stroke or a non-stroke cause (figure 1).

Outcome Assessment

Health outcomes were measured in quality-adjusted life-years (QALYs) which were calculated by multiplying length of life by utility scores derived from the literature.[9 28 29] Utility scores of different disability states after stroke were developed using European quality of life scale (EQ-5D) along with the Chinese preference weights in a Chinese stroke population.[29] In our model, events of recurrent stroke and sICH were considered temporary health states unless they resulted in death. All patients entering these health states were assumed to have a short-term disutility of 30 days for event of recurrent stroke and only 14 days for event of sICH.[9 28] Total economic costs were calculated by multiplying the number of patients in each state by the direct medical costs for that state. The incremental cost-effectiveness ratio (ICER) was calculated by dividing the difference of costs by the difference in QALYs between the two treatment alternatives. We modelled the costs and QALYs gained over the short-term (1, 4, 5 years) and the long-term (30 years). Mechanical thrombectomy was considered cost-effective if the ICER was less than CNY 125,700 (3x GDP per capita of China in 2013,[23] US$ 19,300) per QALY gained. This willingness-to-pay threshold was recommended by the Commission on Macroeconomics and Health of World Health Organization.[26]

Sensitivity Analysis

A deterministic one-way sensitivity analysis, varying the probabilities, utilities and costs, one at a time within the plausible ranges (table 1), was performed to evaluate the uncertainty of the long-term (30 years) results of the model. To determine how much worse mechanical thrombectomy could have performed but still produced a cost-effective ICER, we constructed

For peer review only

two hypothetical worse outcomes for mechanical thrombectomy performance. This was accomplished by setting the odds ratios of mRS 0-2 at day 90 for mechanical thrombectomy treatment at its lower limits of 90% and 95% confidence intervals in the pooled analyses of the 5 trials,[19] representing unfavourable and worse unfavourable scenarios, respectively. For each outcome scenario, we also constructed four hypothetical cost scenarios by setting 10% increase or 10%, 25% and 50% decrease of cost of mechanical thrombectomy.

Furthermore, a probabilistic sensitivity analysis was also undertaken to evaluate the stochastic uncertainty as all variables varied simultaneously. It was performed by using Monte Carlo simulation in Ersatz v1.3 (a bootstrap add-in for Microsoft Excel for Windows; EpiGear International Pty Ltd, Brisbane, Australia). We assumed costs followed a lognormal distribution and the probabilities and utilities followed a beta distribution. The simulation was run 10,000 times. The results were summarized using a scatter-plot and a cost-effectiveness acceptability curve.

Results

Base Case Analysis

Table 2 shows the costs, outcomes and ICER for the mechanical thrombectomy treatment calculated in the short term (1, 4, 5 years) and in the long term (30 years). In the base-case scenario, for a 63-year-old patient with acute ischaemic stroke caused by proximal anterior circulation occlusion within 6 hours after onset of stroke, mechanical thrombectomy would be cost-ineffective in the first four years, but become cost-effective from the fifth year onwards, using the threshold of CNY 125,700 (3x GDP per capita of China in 2013, US$ 19,300) as the willingness-to-pay per QALY. After 5 years, mechanical thrombectomy gained 0.457 QALYs at

For peer review only

an additional cost of CNY 52,300 (US$ 8,050), yielding an ICER of CNY 114,500 (US$ 17,620) per QALY gained. In the long term (30 years), mechanical thrombectomy gained 0.930 QALYs at an additional cost of CNY 54,600 (US$ 8,400), yielding an ICER of CNY 58,700 (US$ 9,030) per QALY gained.

Sensitivity Analysis

The results of the deterministic one-way sensitivity analysis for the ICER of mechanical thrombectomy in the long term are showed in the tornado diagrams (figure 2). Overall, the ICER was most sensitive to the additional cost of mechanical thrombectomy, odds ratio of favourable functional outcome (mRS 0-2) at day 90 and annual post-hospitalization costs for patients with independence. Even if the additional cost of mechanical thrombectomy increased to CNY 91,094, the ICER of mechanical thrombectomy (CNY 87,355/QALY) was still within the threshold of the willingness-to-pay per QALY (CNY 125,700, 3x GDP per capita of China in 2013). In each hypothetical case scenario, mechanical thrombectomy continued to produce a benefit in QALYs (online supplementary table 1). Even in the worst hypothetical scenario with worse unfavourable effect outcome and an increase of 10% price of mechanical thrombectomy, mechanical thrombectomy continued to be cost-effective (ICER: CNY 91,240/QALY). In the hypothetical case scenario with base case effect outcome and a decrease of 50% price of mechanical thrombectomy, mechanical thrombectomy could be high cost-effective (ICER: CNY 29,935/QALY<CNY 41,900 [1x GDP per capita of China in 2013, US$ 6,400]/QALY).

Figure 3 shows the results of the probabilistic sensitivity analysis in the long term. Among the 10,000 simulation runs, mechanical thrombectomy was cost-effective in 95.4% of the simulations at a willingness-to-pay threshold of CNY 125,700 (3x GDP per capita of China in 2013, US$ 19,300) per QALY, and in 28.7% of the simulations at a threshold of CNY 41,900

For peer review only - http://bmjopen.bmj.com/site/about/guidelines.xhtml

(1x GDP per capita of China in 2013, US$ 6,400) per QALY. Online supplementary figure 1 shows the cost-effectiveness acceptability curve of mechanical thrombectomy.

Discussion

Our study indicated that mechanical thrombectomy with second generation devices for acute ischaemic stroke caused by proximal anterior circulation occlusion within 6 hours after onset of stroke was cost-effective from the fifth year onwards in China. A patient with acute ischaemic stroke treated with mechanical thrombectomy gain an ICER of CNY 58,700 per QALY in the long term, which was below 3x GDP per capita of China in 2013 (CNY 125,700). We also found that the ICER is more sensitive to additional cost of mechanical thrombectomy, odds ratio of favourable functional outcome at day 90 and annual post-hospitalization costs for patients with independence.

Similar as the results of cost-effective completed in other high-income countries,[7-10] our study shows that mechanical thrombectomy treatment is also cost-effective in low- and middle-income countries like China. The lifetime gain of 0.93 QALYs for mechanical thrombectomy treatment for acute ischaemic stroke in our study was comparable to that evaluated in the high income countries (0.7 QALYs in the USA,[7] 1.05 QALYs in the UK[9] and 0.99 QALYs in Sweden[10]). The QALYs gain of mechanical thrombectomy treatment was relatively more than that of most of other treatments for stroke. For example, the lifetime QALYs gain was 0.42 for IV tPA treatment for acute ischaemic stroke within 4.5 hours,[30] and 0.17 for clopidogrel for secondary prevention of stroke comparing with aspirin.[31] This may be mainly because of the large magnitude of effect of mechanical thrombectomy (odds ratio 2.35).[19]

For peer review only

Mechanical thrombectomy treatment with second generation devices has been accepted as the standard of care for acute ischaemic stroke patients caused by proximal anterior circulation occlusions within 6 hours after symptom onset.[32 33] However, economic costs of mechanical thrombectomy treatment are extremely high, especially in low- and middle-income countries like China, where people suffer higher burden of stroke and intracranial atherosclerosis is much more prevalent than the Western countries.[34] The additional costs of mechanical thrombectomy is approximately 5-fold of one-time hospitalization costs without tPA treatment and mechanical thrombectomy in China, comparing with only 1.5-fold in the USA.[7 8] Previous studies showed that there were socioeconomic disparities in the utilization of mechanical thrombectomy for acute ischaemic stroke and patients with low income or in remote areas had lower rates of mechanical thrombectomy utilization.[35 36] The implement of mechanical thrombectomy treatment are to-some-extent depend on the cost-effectiveness of the technology, which is of importance for the clinical decision in the low- and middle-income countries. Our study supports the implement of mechanical thrombectomy treatment after acute ischaemic stroke in clinical practice in the low- and middle-income countries from the perspective of economics, and may provide an important reference for the low income or remote areas in the Western countries.

Proximal large vessel atherosclerotic stenosis or occlusion accounts for 35–40% of all acute ischaemic strokes,[17] among which about 40% arrived at the hospital within 6 hours in China.[18] Therefore, about 14%-16% of ischaemic stroke patients were eligible mechanical thrombectomy and might benefit from this procedure. However, the real-world implement of endovascular thrombectomy treatment in low- and middle-income countries and areas may be restricted by the poor awareness of the public, poor infrastructure, inefficient system, deficiency of specialists and the timeliness of patients got to the hospital (within 6 hours), which may cause inequity for those

For peer review only

who cannot receive the technology.[10] To generalize this new technology in these countries and areas, education targeted to the public, hospital administration and governmental agencies should be improved so that they can better understand the benefit and cost-effectiveness of thrombectomy.[37] Furthermore, service system redesign is needed to establish efficient care chains and workflow with coordination among neurointerventinalists and other departments. Additionally, high experience and skills are required to perform this advanced technology, while ways of providing interventional treatment are unstandardized and quite diverse in low- and middle- income countries like China.[37] Rapid efforts are urgently required to organize standardized training of performing thrombectomy to handle deficiency of intervention specialists in these countries and areas.

This study has several limitations that should be considered when interpreting the results. First, our model focused on the impact of mechanical thrombectomy treatment on acute ischaemic stroke, and health status and costs as a result of other causes, such as occurrence of intracranial haemorrhage and myocardial infarction, were not included in this model. Second, costs of transfer to the hospitals doing mechanical thrombectomy has not been included in this analysis. Third, functional improvement after rehabilitation was not considered in the model for lack of data available on the efficacy of rehabilitation. However, organized rehabilitation after stroke in China is poor.[38 39] Fourth, we to some extent arbitrarily assumed that the patients with independent state remaining alive after stroke recurrence were equally reallocated in the categories of independent and dependent state. However, this is not unprecedented in the modelling of cost-effectiveness analysis for stroke.[21] Finally, the efficacy of mechanical thrombectomy treatment was based on trials completed in high income countries with older age of patients than that in China. Additionally, most of the trials ceased early, all were sponsored by

For peer review only

industry which may cause potential risk of bias, and some of the patients included in the trials did not received IV tPA treatment. However, this is the only data available. These limitations would have led to under- or over- estimation of the true cost-effectiveness of mechanical thrombectomy treatment in low- and middle-income countries.

Conclusions

Mechanical thrombectomy with second generation devices for acute ischaemic stroke caused by proximal anterior circulation occlusion within 6 hours after onset of stroke was cost-effective in China. Our study supports the implement of mechanical thrombectomy treatment after acute ischaemic stroke in clinical practice in low- and middle-income countries, and may also be a reference to the low income or remote areas in the developed countries. More medical resources related to mechanical thrombectomy should be allocated in these areas.

Contributors

YSP, YJW and YLW designed the study and drafted the manuscript. YSP, XLC and XCH: collected the data, performed the literature search and constructed the decision tree. XQZ, LPL and ZRM interpreted the data and revised the manuscript.

Funding

This work was supported by grants from the Ministry of Science and Technology of the People's Republic of China (2015BAI12B04, 2015BAI12B02, 2016YFC0901000, 2016YFC0901001, 2016YFC0901002), grants from Beijing Municipal Science and Technology Commission (Z15110200390000, Z151100003915117), and grants from Beijing Municipal Commission of Health and Family Planning (No.2016-1-2041, SML20150502).

For peer review only

Competing interests

None declared.

Data sharing statement

We, as the authors of this original research article, state that there are no additional, unpublished data available from this study.

For peer review only

References

1. Lees KR, Bluhmki E, von Kummer R, *et al*. Time to treatment with intravenous alteplase and outcome in stroke: an updated pooled analysis of ECASS, ATLANTIS, NINDS, and EPITHET trials. *Lancet* 2010;375:1695-703.
2. Berkhemer OA, Fransen PS, Beumer D, *et al*. A randomized trial of intraarterial treatment for acute ischemic stroke. *N Engl J Med* 2015;372:11-20.
3. Goyal M, Demchuk AM, Menon BK, *et al*. Randomized assessment of rapid endovascular treatment of ischemic stroke. *N Engl J Med* 2015;372:1019-30.
4. Saver JL, Goyal M, Bonafe A, *et al*. Stent-retriever thrombectomy after intravenous t-PA vs. t-PA alone in stroke. *N Engl J Med* 2015;372:2285-95.
5. Jovin TG, Chamorro A, Cobo E, *et al*. Thrombectomy within 8 hours after symptom onset in ischemic stroke. *N Engl J Med* 2015;372:2296-306.
6. Campbell BC, Mitchell PJ, Kleinig TJ, *et al*. Endovascular therapy for ischemic stroke with perfusion-imaging selection. *N Engl J Med* 2015;372:1009-18.
7. Leppert MH, Campbell JD, Simpson JR, Burke JF. Cost-Effectiveness of Intra-Arterial Treatment as an Adjunct to Intravenous Tissue-Type Plasminogen Activator for Acute Ischemic Stroke. *Stroke* 2015;46:1870-6.
8. Kunz WG, Hunink MG, Sommer WH, *et al*. Cost-Effectiveness of Endovascular Stroke Therapy: A Patient Subgroup Analysis From a US Healthcare Perspective. *Stroke* 2016;47:2797-804.
9. Ganesalingam J, Pizzo E, Morris S, Sunderland T, Ames D, Lobotesis K. Cost-Utility Analysis of Mechanical Thrombectomy Using Stent Retrievers in Acute Ischemic Stroke. *Stroke* 2015;46:2591-8.

For peer review only

10. Aronsson M, Persson J, Blomstrand C, Wester P, Levin LÅ. Cost-effectiveness of endovascular thrombectomy in patients with acute ischemic stroke. *Neurology* 2016;86:1053-9.
11. Lobotesis K, Veltkamp R, Carpenter IH, Claxton LM, Saver JL, Hodgson R. Cost-effectiveness of Stent-retriever Thrombectomy in combination with IV t-PA Compared with IV t-PA Alone for Acute Ischemic Stroke in the UK. *J Med Econ* 2016:1-37.
12. Steen Carlsson K, Andsberg G, Petersson J, Norrving B. Long-term cost-effectiveness of thrombectomy for acute ischaemic stroke in real life: An analysis based on data from the Swedish Stroke Register (Riksstroke). *Int J Stroke* 2017:1747493017701154.
13. de Andrés-Nogales F, Álvarez M, de Miquel MÁ, et al. Cost-effectiveness of mechanical thrombectomy using stent retriever after intravenous tissue plasminogen activator compared with intravenous tissue plasminogen activator alone in the treatment of acute ischaemic stroke due to large vessel occlusion in Spain. *Eur Stroke J* 2017;2:272-84.
14. Shireman TI, Wang K, Saver JL, et al. Cost-Effectiveness of Solitaire Stent Retriever Thrombectomy for Acute Ischemic Stroke: Results From the SWIFT-PRIME Trial (Solitaire With the Intention for Thrombectomy as Primary Endovascular Treatment for Acute Ischemic Stroke). *Stroke* 2017;48:379-87.
15. Xie X, Lambrinos A, Chan B, et al. Mechanical thrombectomy in patients with acute ischemic stroke: a cost-utility analysis. *CMAJ Open* 2016;4:E316-25.
16. Yang G, Wang Y, Zeng Y, *et al*. Rapid health transition in China, 1990-2010: findings from the Global Burden of Disease Study 2010. *Lancet* 2013;381:1987-2015.

For peer review only

17. Miao Z, Huo X, Gao F, *et al*. Endovascular therapy for Acute ischemic Stroke Trial (EAST): study protocol for a prospective, multicentre control trial in China. *Stroke Vasc Neurol* 2016;1:44-51.

18. Wang Y, Cui L, Ji X, *et al*. The China National Stroke Registry for patients with acute cerebrovascular events: design, rationale, and baseline patient characteristics. *Int J Stroke* 2011;6:355-61.

19. Goyal M, Menon BK, van Zwam WH, *et al*. Endovascular thrombectomy after large-vessel ischaemic stroke: a meta-analysis of individual patient data from five randomised trials. *Lancet* 2016;387:1723-31.

20. Pennlert J, Eriksson M, Carlberg B, Wiklund PG. Long-term risk and predictors of recurrent stroke beyond the acute phase. *Stroke* 2014;45:1839-41.

21. Tung CE, Win SS, Lansberg MG. Cost-effectiveness of tissue-type plasminogen activator in the 3- to 4.5-hour time window for acute ischemic stroke. *Stroke* 2011;42:2257-62.

22. National Bureau of Statistics of China. The 2010 population census of the peaple's republic of china. Websit: http://www.stats.gov.cn/tjsj/pcsj/rkpc/6rp/indexch.htm (in Chinese). [Accessed Feb 25, 2016].

23. Ministry of Health of the People's Republic of China. China Health Statistics Yearbook 2014. Peking Union Medical College Press. Beijing (2014) (in Chinese).

24. Samsa GP, Reutter RA, Parmigiani G, *et al*. Performing cost-effectiveness analysis by integrating randomized trial data with a comprehensive decision model: application to treatment of acute ischemic stroke. *J Clin Epidemiol* 1999;52:259-71.

25. Liao XL, Wang CX, Wang YL, *et al*. Implementation and outcome of thrombolysis with alteplase 3 to 4.5 h after acute stroke in Chinese patients. *CNS Neurosci Ther* 2013;19:43-7.

For peer review only

26. Pan Y, Wang A, Liu G, *et al*. Cost-effectiveness of clopidogrel-aspirin versus aspirin alone for acute transient ischemic attack and minor stroke. *J Am Heart Assoc* 2014;3:e000912.

27. Kim AS, Nguyen-Huynh M, Johnston SC. A cost-utility analysis of mechanical thrombectomy as an adjunct to intravenous tissue-type plasminogen activator for acute large-vessel ischemic stroke. *Stroke* 2011;42:2013-8.

28. Lee S, Anglade MW, Pham D, Pisacane R, Kluger J, Coleman CI. Cost-effectiveness of rivaroxaban compared to warfarin for stroke prevention in atrial fibrillation. *Am J Cardiol* 2012;110:845-51.

29. Wang YL, Pan YS, Zhao XQ, *et al*. Recurrent stroke was associated with poor quality of life in patients with transient ischemic attack or minor stroke: finding from the CHANCE trial. *CNS Neurosci Ther* 2014;20:1029-35.

30. Pan Y, Chen Q, Zhao X, *et al*. Cost-Effectiveness of Thrombolysis within 4.5 Hours of Acute Ischemic Stroke in China. *PLoS One* 2014;9:e110525.

31. Schleinitz MD, Weiss JP, Owens DK. Clopidogrel versus aspirin for secondary prophylaxis of vascular events: a cost-effectiveness analysis. *Am J Med* 2004;116:797-806.

32. Powers WJ, Derdeyn CP, Biller J, *et al*. 2015 American Heart Association/American Stroke Association Focused Update of the 2013 Guidelines for the Early Management of Patients With Acute Ischemic Stroke Regarding Endovascular Treatment: A Guideline for Healthcare Professionals From the American Heart Association/American Stroke Association. *Stroke* 2015;46:3020-35.

33. Miao Z, Wang Y. Chinese guidelines for endovascular treatment for acute ischemic stroke 2015.(in Chinese). *Chin J Stroke* 2015;10:590-606.

For peer review only

34. Wang Y, Zhao X, Liu L, *et al*. Prevalence and outcomes of symptomatic intracranial large artery stenoses and occlusions in China: the Chinese Intracranial Atherosclerosis (CICAS) Study. *Stroke* 2014;45:663-9.

35. Brinjikji W, Rabinstein AA, Cloft HJ. Socioeconomic disparities in the utilization of mechanical thrombectomy for acute ischemic stroke. *J Stroke Cerebrovasc Dis* 2014;23:979-84.

36. Pérez de la Ossa N, Abilleira S, Dorado L, *et al*. Access to Endovascular Treatment in Remote Areas: Analysis of the Reperfusion Treatment Registry of Catalonia. *Stroke* 2016;47:1381-4.

37. Miao Z. New era of stroke therapy and new challenges. *Stroke Vasc Neurol* 2016;1:6-7.

38. Li AD, Huang ZQ, Liu HT, Deng YT, Xiao JW, Liang JW. Knowledge, Attitude and Behavior for Stroke and Rehabilitation in Stroke Patients and Relatives.(in Chinese). *Chin J Rehabil Theory Pract* 2009;15:252-4.

39. Gao YL, Chen LD, Tao J, Chen SM. Investigation on the investment and popularization of rehabilitation from cerebral apoplexy: in 2 communities of Xiamen City.(in Chinese). *Chin J Convalescent Med* 2009;18:472-4.

For peer review only

Figure Legends

Figure 1. Decision tree and Markov state transition model. A patient with an acute ischaemic stroke with anterior circulation occlusion entered the model at 63 years old receiving either intravenous tissue-type plasminogen activator (IV tPA) with or without mechanical thrombectomy, and transited between health states until death or 30 years. Patients may remain in the same health state, move to a state of equal or greater disability after recurrent stroke, or die. Only transition from dependent state (modified Rankin Scale [mRS] 3-5) was illustrated in the figure. M indicates Markov node; sICH, symptomatic intracerebral haemorrhage.

Figure 2. One-way sensitivity analysis on incremental cost-effectiveness ratio (ICER) gained in the long term (30 years) by mechanical thrombectomy. All model input parameters were analysed, and only ten parameters with the highest relative effects on ICER are displayed. Base-case scenario of ICER is CNY 58,710 per quality-adjusted life-year (QALY) gained.

Figure 3. Scatterplot of the result of probabilistic sensitivity analysis in the long term (30 years). Each point represents a simulation. The dark square represents the base case (0.930 QALYs gained at an incremental cost of CNY 54,600). The solid line represents the willingness-to-pay threshold of CNY 125,700 per QALY. The dashed line represents CNY 41,900 per QALY. Points to the right of the solid line are considered cost-effective.

For peer review only - http://bmjopen.bmj.com/site/about/guidelines.xhtml

Tables

Table 1. Base-case and Plausible Ranges of Model Inputs

Model Input	Base Case	Range	Reference
**Efficacy and safety outcome inputs**			[19]
Proportion of outcomes at 90 days in IV tPA group			
mRS 0-2	0.265	0.231-0.301	
Death (mRS 6)	0.189	0.159-0.221	
sICH	0.043	0.029-0.061	
Odds ratio at 90 days			
mRS 0-2	2.35	1.85-2.98	
Death (mRS 6)	0.77	0.54-1.10	
sICH	1.07	0.62-1.83	
**Probabilities inputs**			
Proportion of patients received mechanical thrombectomy	0.880	0.852-0.904	[2-6]
Proportion of patients arrived within 4.5 hours	0.854	0.839-0.869	CNSR
Recurrent rate of stroke (per patient year)			CNSR
mRS 0-2	0.1026	0.0961-0.1093	
mRS 3-5	0.1418	0.1303-0.1534	
Relative risk of stroke recurrence per life year	1.03	1.02-1.04	[20]
Death with recurrent stroke	0.2101	0.1887-0.2316	CNSR
Age specific non-stroke death rate *	0.0089-0.1653		[22 23]

For peer review only

Hazard ratio of non-stroke death for mRS 3-5	1.78	1.43-2.14	[24]
**Cost inputs (2013 Chinese Yuan Renminbi)**			
Additional costs of mechanical thrombectomy	60821	48525-91094	EAST
Additional costs of IV tPA treatment	11179	9446-13336	CNSR, TIMS-CHINA
Additional costs of sICH	2374	516-4955	TIMS-CHINA
One-time hospitalization costs			CNSR
mRS 0-2	10055	3941-17732	
mRS 3-5	13729	4627-25590	
mRS 6	11121	3322-21859	
Annual post-hospitalization costs			CNSR
mRS 0-2	7385	556-17801	
mRS 3-5	11350	1335-26703	
**Utility inputs**			
mRS 0-2	0.76	0.69-0.82	[29]
mRS 3-5	0.21	0.17-0.26	[29]
Death (mRS 6)	0	0.00-0.00	[29]
Recurrent stroke	0.34	0.32-0.36	[9]
sICH	0.84	0.72-1.0	[28]
**Discount rate inputs**			
Costs	0.03	0.03-0.08	[26]
Outcomes	0.03	±20%	[26]

For peer review only

All costs were converted to 2013 Chinese Yuan Renminbi by using the medical care component of consumer price index; to convert CNY to US dollars, divide by 6.5.

IV tPA, intravenous tissue plasminogen activator; mRS, modified Rankin Scale; sICH, symptomatic intracerebral haemorrhage; CNSR, China National Stroke Registry; EAST, Endovascular therapy for Acute ischaemic Stroke Trial; TIMS-CHINA, Thrombolysis Implementation and Monitor of acute ischaemic Stroke in China.

* Age specific non-stroke death rate: only the number of 63 years old (0.0089) and 93 years old (0.1653) are presented.

For peer review only

Table 2. Costs and Outcomes in Base-case Analysis

Time horizon	Treat strategy	QALYs	Cost (CNY)	ICER (CNY/QALY)
1 year	IV tPA alone	0.373	30,810	-
	Mechanical thrombectomy + IV tPA	0.494	83,940	439,091
4 years	IV tPA alone	1.106	55,200	-
	Mechanical thrombectomy + IV tPA	1.492	107,630	135,829
5 years	IV tPA alone	1.307	62,260	-
	Mechanical thrombectomy + IV tPA	1.764	114,590	114,508
30 years	IV tPA alone	2.684	119,620	-
	Mechanical thrombectomy + IV tPA	3.614	174,220	58,710

IV tPA, intravenous tissue plasminogen activator; QALY, quality-adjusted life-year; ICER, incremental cost-effectiveness ratio.

review only

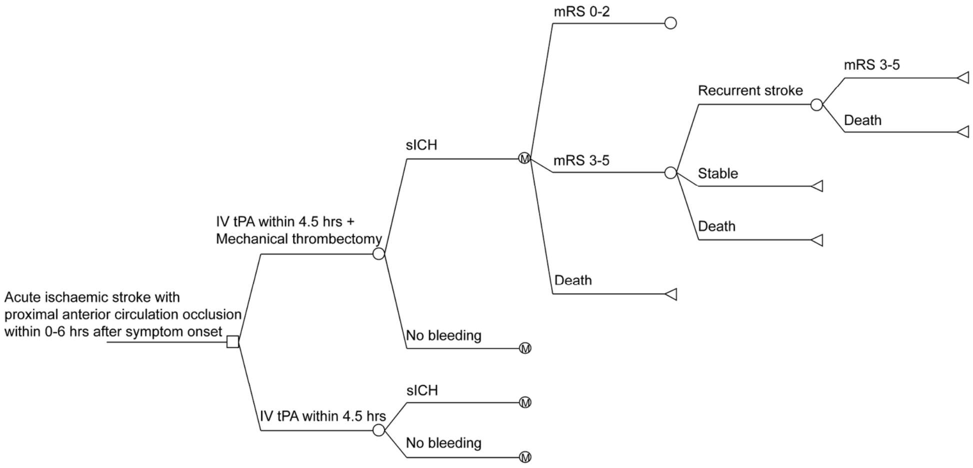

Figure 1. Decision tree and Markov state transition model. A patient with an acute ischaemic stroke with anterior circulation occlusion entered the model at 63 years old receiving either intravenous tissue-type plasminogen activator (IV tPA) with or without mechanical thrombectomy, and transited between health states until death or 30 years. Patients may remain in the same health state, move to a state of equal or greater disability after recurrent stroke, or die. Only transition from dependent state (modified Rankin Scale [mRS] 3-5) was illustrated in the figure. M indicates Markov node; sICH, symptomatic intracerebral haemorrhage.

84x41mm (600 x 600 DPI)

view only

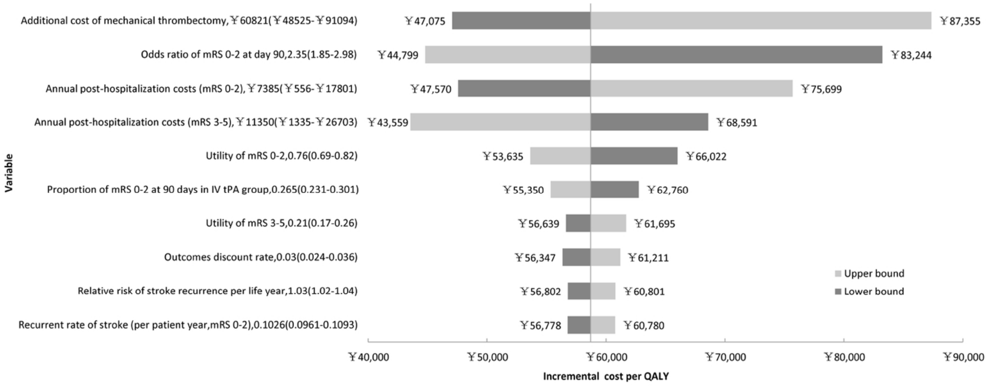

Figure 2. One-way sensitivity analysis on incremental cost-effectiveness ratio (ICER) gained in the long term (30 years) by mechanical thrombectomy. All model input parameters were analysed, and only ten parameters with the highest relative effects on ICER are displayed. Base-case scenario of ICER is CNY 58,710 per quality-adjusted life-year (QALY) gained.

68x27mm (600 x 600 DPI)

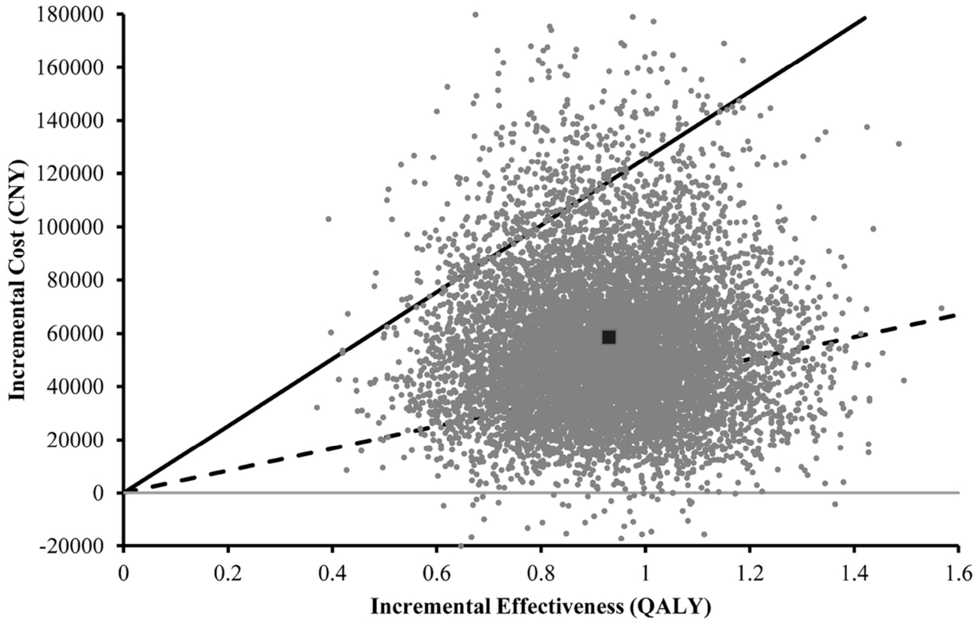

Figure 3. Scatterplot of the result of probabilistic sensitivity analysis in the long term (30 years). Each point represents a simulation. The dark square represents the base case (0.930 QALYs gained at an incremental cost of CNY 54,600). The solid line represents the willingness-to-pay threshold of CNY 125,700 per QALY. The dashed line represents CNY 41,900 per QALY. Points to the right of the solid line are considered cost-effective.

55x36mm (600 x 600 DPI)

ONLINE SUPPLEMENTARY MATERIALS

Table 1. Hypothetical Worse Performance and Costs of Mechanical Thrombectomy, ICER (CNY/QALY)

Additional cost of mechanical thrombectomy	Outcome of mechanical thrombectomy: Base Case (OR=2.35)*	Unfavourable (OR=1.93)	Worse unfavourable (OR=1.85)
+10%, CNY 66903	64,462	85,224	91,241
Base case, CNY 60821	58,710	77,731	83,244
-10%, CNY 54739	52,957	70,238	75,247
-25%, CNY 45616	44,323	58,992	63,244
-50%, CNY 30410	29,935	40,252	43,244

ICER, incremental cost-effectiveness ratio; QALY, quality-adjusted life-year; OR, Odds ratio.

* OR: OR of modified Rankin Scale 0-2 at day 90 for mechanical thrombectomy.

For peer review only

Figure 1. Cost-effectiveness acceptability curve. The curve presents the probability that mechanical thrombectomy within 6 hours to be cost-effective against willingness-to-pay threshold. The solid line represents the willingness-to-pay threshold of CNY 125,700 per QALY. The dashed line represents CNY 41,900 per QALY.

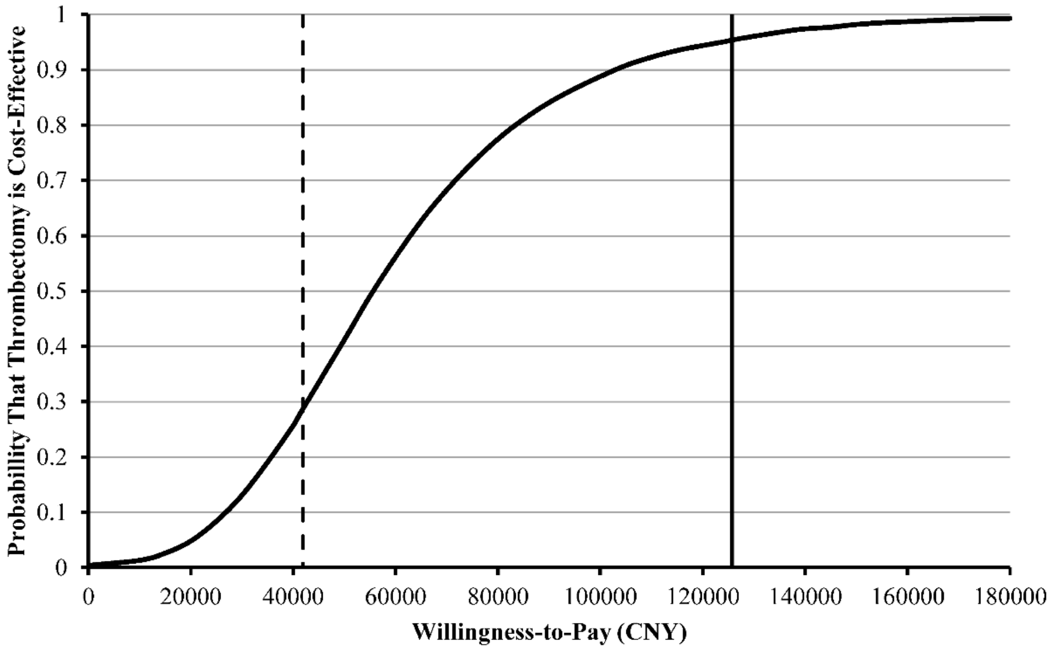

BMJ Open

Cost-Effectiveness of Mechanical Thrombectomy within 6 Hours of Acute Ischaemic Stroke in China

Journal:	*BMJ Open*
Manuscript ID	bmjopen-2017-018951.R2
Article Type:	Research
Date Submitted by the Author:	29-Dec-2017
Complete List of Authors:	PAN, YUESONG; Beijing Tiantan Hospital, Capital Medical University, Department of Neurology<br>Cai, Xueli; Lishui Hospital of Zhejiang University, Department of Neurology<br>Huo, Xiaochuan; Beijing Tiantan Hospital, Interventional Neurology;<br>Zhao, Xingquan; Beijing Tiantan Hospital, Neurology<br>Liu, Liping; Beijing Tiantan Hospital, Capital Medical University, Department of Neurology<br>Wang, Yongjun; Beijing Tiantan Hospital, Neurology<br>Miao, Zhongrong; Beijing Tiantan Hospital, Neurology<br>Wang, Yilong; Beijing Tiantan Hospital, Neurology
**Primary Subject Heading**:	Health economics
Secondary Subject Heading:	Neurology
Keywords:	cost-effectiveness, costs, quality-adjusted life-year, Stroke < NEUROLOGY, thrombectomy

SCHOLARONE™ Manuscripts

For peer review only

Cost-Effectiveness of Mechanical Thrombectomy within 6 Hours of Acute Ischaemic Stroke in China

Cover title: CEA of mechanical thrombectomy of ischaemic stroke

Yuesong Pan,[1,2,3,4,5*] Xueli Cai,[6*] Xiaochuan Huo,[1,2,3,4] Xingquan Zhao,[1,2,3,4] Liping Liu,[1,2,3,4] Yongjun Wang,[1,2,3,4] Zhongrong Miao,[1,2,3,4] Yilong Wang[1,2,3,4]

[1] Department of Neurology, Beijing Tiantan Hospital, Capital Medical University, Beijing, China

[2] China National Clinical Research Centre for Neurological Diseases, Beijing, China

[3] Centre of Stroke, Beijing Institute for Brain Disorders, Beijing, China

[4] Beijing Key Laboratory of Translational Medicine for Cerebrovascular Disease, Beijing, China

[5] Department of Epidemiology and Health Statistics, School of Public Health, Capital Medical University, Beijing, China.

[6] Department of Neurology, Lishui Hospital of Zhejiang University (the Central Hospital of Lishui), Lishui, Zhejiang, China.

* Yuesong Pan and Xueli Cai contributed equally to the manuscript.

Corresponding author: Yilong Wang

Address: No.6 Tiantanxili, Dongcheng District, Beijing, China, 100050

Tel: 0086-010-67092222

Fax: 0086-010-67098542

E-mail: yilong528@gmail.com

Co-corresponding author: Zhongrong Miao **E-mail:** zhongrongm@163.com

Tables: 2; **Figure:** 3; **Supplemental table:** 2; **Supplemental figure:** 1; **Words:** 3298.

Key Words: cost-effectiveness; costs; quality-adjusted life-year; stroke; thrombectomy.

For peer review only

Abstract

Objectives Endovascular mechanical thrombectomy is an effective but expensive therapy for acute ischaemic stroke with proximal anterior circulation occlusion. This study aimed to determine the cost-effectiveness of mechanical thrombectomy in China, the largest developing country.

Design A combination of decision tree and Markov model was developed. Outcome and cost data were derived from the published literature and claims database. The efficacy data were derived from the meta-analyses of 9 trials. One-way and probabilistic sensitivity analyses were performed in order to assess the uncertainty of the results.

Setting Hospitals in China.

Participants The patients with acute ischaemic stroke caused by proximal anterior circulation occlusion within 6 h.

Interventions Mechanical thrombectomy within 6 h with IV tPA treatment within 4.5 h versus IV tPA treatment alone.

Outcome measures The benefit conferred by the treatment was assessed by estimating the cost per quality-adjusted life-year (QALY) gained in the long term (30 years).

Results The addition of mechanical thrombectomy to IV tPA treatment compared with standard treatment alone yielded a lifetime gain of 0.794 QALYs at an additional cost of CNY 50,000 (US$ 7,700), resulting in a cost of CNY 63,010 (US$ 9,690) per QALY gained. The probabilistic sensitivity analysis indicated that mechanical thrombectomy was cost-effective in 99.9% of the simulation runs at a willingness-to-pay threshold of CNY 125,700 (US$ 19,300) per QALY.

Conclusions Mechanical thrombectomy for acute ischaemic stroke caused by proximal anterior circulation occlusion within 6 h was cost-effective in China. The data may be used as a reference

For peer review only

with regard to medical resources allocation for stroke treatment in low- and middle-income countries as well as in the remote areas in the developed countries.

For peer review only

Strengths and limitations of this study

- A combination of decision tree and Markov model was developed in order to simulate the short-term and long-term costs and outcomes after mechanical thrombectomy for ischaemic stroke.
- The majority of the parameters used in the model, including costs, utilities and transition probabilities, were collected based on the Chinese setting, reflecting the situation in the low- and middle-income countries.
- A limitation of the present study is that the health status and costs that resulted from additional causes other than stroke were not included in this model.
- An additional limitation is that the efficacy of the mechanical thrombectomy treatment was based on trials that were completed in high income countries; however, these were the only data available.

For peer review only

Background

Arterial recanalisation and subsequent reperfusion performed shortly after acute ischaemic stroke have demonstrated their ability to restore brain function.[1] Besides intravenous recombinant tissue-type plasminogen activator (IV tPA) within 4.5 h, endovascular mechanical thrombectomy is another effective reperfusion strategy, which can remove large, proximal clots rapidly and results in higher rate of reperfusion compared with IV tPA alone.[2] Second-generation retrievable stents can achieve higher recanalisation rates compared with first-generation devices. Although trials that have used first-generation thrombectomy devices failed to demonstrate clinical benefit compared with IV tPA, the five recently published clinical trials in 2015 that included second-generation devices, have shown clear benefits with regard to the addition of mechanical thrombectomy to standard treatment for acute ischaemic stroke caused by proximal anterior circulation occlusions.[2-6]

Although mechanical thrombectomy with second generation devices exhibits optimal effectiveness with acceptable safety, the main disadvantage is the high cost that requires expensive devices, highly trained proceduralists and special periprocedural support. Previous studies that have examined the economic aspects of this type of therapy were conducted in the USA, UK, Sweden, Canada and Spain. These studies indicated that mechanical thrombectomy treatment for acute ischaemic stroke was cost-effective or even cost-saving in the long term.[7-15] However, all of these studies were conducted in high income countries and the corresponding results may not be applicable in low- and middle-income countries such as China, where medical resources are scarce and stroke is a leading cause of death.[16] Little is known with regard to the cost-effectiveness and feasibility of the application of mechanical thrombectomy treatment in patients with acute ischaemic stroke in low- and middle-income countries. The analysis of the

For peer review only

economic costs involved in mechanical thrombectomy in low- and middle-income countries is urgent. In the present study, we aimed to evaluate the cost-effectiveness of the addition of mechanical thrombectomy treatment with second generation devices to standard care for acute ischaemic stroke in the setting of China, which is considered the largest low- and middle-income country.

Methods

Model Overview

A combination of decision tree and Markov model (figure 1) was developed in order to simulate the short-term (1, 5, 6 years) and long-term (30 years) cost-effectiveness of mechanical thrombectomy using stent retrievers within 6 h with IV tPA treatment within 4.5 h versus IV tPA treatment alone within 4.5 h after onset of stroke. The base case of the model was a cohort of 100,000 patients with acute ischaemic stroke with proximal anterior circulation occlusion (36% female), with mean age of 63 years old, being admitted to the hospital within 6 h after onset of stroke. The baseline characteristics were the same as patients enrolled in the treated arm of the Endovascular therapy for Acute ischaemic Stroke Trial (EAST, a non-randomized interventional study that aimed to evaluate the safety and efficacy of Solitaire thrombectomy in Chinese patients with acute stroke),[17] who were admitted to the hospital within 6 h. Among the patients admitted to the hospital within 6 h after the onset of stroke, we assumed that 85.4% patients were admitted within 4.5 h in both arms according to the data from the China National Stroke Registry (CNSR, a nationwide registry that has recruited 21,902 consecutive patients with acute cerebrovascular events from 132 hospitals in China between September 2007 and August 2008.),[18] all of whom were assumed to receive IV tPA treatment; while all patients in the

For peer review only

intervention arm received mechanical thrombectomy treatment. The total costs and quality-adjusted life-years (QALYs) that were gained with each alternative type of treatment were estimated for each health state at 90 days from the index event, at a 9 months period following this time point and then estimated annually for the remaining 30 years. The half cycle correction was conducted for the years spent in the corresponding states that were subsequently used to calculate the health outcomes and costs. The transition probability and costs were discounted for the first 3 months segment and for the next 9 months segment in the first year. This analysis was conducted from the perspective of healthcare payers. The present study used published data and anonymised clinical data of patients from databases and therefore was exempt from institutional review board approval.

Input Parameters

The model input parameters were drawn from the published literature and the claims database (table 1). The proportion of efficacy and safety outcomes at 90 days in the IV tPA group was estimated by meta-analysis based on a random effect model (I^2>50%) of the recently published 9 trials, including the Multicentre Randomized Clinical Trial of Endovascular Treatment for Acute Ischaemic Stroke in the Netherlands (MR CLEAN),[2] the Endovascular Treatment for Small Core and Anterior Circulation Proximal Occlusion with Emphasis on Minimizing CT to Recanalisation Times (ESCAPE),[3] the Randomized Trial of Revascularization with Solitaire FR Device versus Best Medical Therapy in the Treatment of Acute Stroke Due to Anterior Circulation Large Vessel Occlusion Presenting within Eight Hours of Symptom Onset (REVASCAT),[4] the Extending the Time for Thrombolysis in Emergency Neurological Deficits-Intra-Arterial (EXTEND IA),[5] the Solitaire with the Intention for Thrombectomy as Primary Endovascular Treatment (SWIFT PRIME),[6] the THRombectomie des Artères CErebrales

For peer review only

(THRACE),[19] The Randomized, Concurrent Controlled Trial to Assess the Penumbra System's Safety and Effectiveness in the Treatment of Acute Stroke (THERAPY),[20] the Pragmatic Ischaemic Thrombectomy Evaluation (PISTE)[21] and the Endovascular Acute Stroke Intervention (EASI)[22]. The efficacy and safety outcomes (odds ratios [ORs]) of mechanical thrombectomy-treated patients at day 90 were estimated by meta-analysis based on a fixed effect model (I^2<50%) of the aforementioned 9 trials (table 1). Subsequently, the proportions of the outcomes in the mechanical thrombectomy-treated groups were calculated based on the proportions in the IV tPA group and the ORs for the outcomes measured, as determined by the following formula: p2=(OR*p1)/(1+(OR-1)*p1).

The recurrent rates of stroke by modified Rankin Scale (mRS) categories and the death rate with recurrent strokes in years following the first 90 days were estimated from the CNSR study.[18] We further assumed an increase in the risk of stroke recurrence by 1.03-fold per life year.[23] The patients who remained alive after stroke recurrence were assumed to be reallocated equally among categories of equal and greater disability.[24] This indicated that patients in the independent state (mRS 0-2) who had a recurrent stroke and survived were allocated equally among the independent (mRS 0-2) and the dependent states (mRS 3-5); while patients in the dependent state remaining alive after stroke recurrence were all remain in dependent state.

The age specific non-stroke death rates were derived from the most recent published census of China and were adjusted according to the causes of death of 2013 reported in the China Health Statistics Yearbook 2014.[25 26] The disability status was assumed to affect the survival rate, and therefore the final age specific non-stroke death rates for those with dependent state were adjusted by the hazard ratio of death for mRS 3-5.[27]

Costs

For peer review only - http://bmjopen.bmj.com/site/about/guidelines.xhtml

The total costs including both out-of-pocket costs and reimbursements, were converted to 2013 Chinese Yuan Renminbi (CNY) using the medical care component of consumer price index.[26] The average cost of one-time hospitalisation and annual post-hospitalisation costs (such as inpatient and outpatient rehabilitation and secondary preventive costs) after stroke according to the categories of mRS were obtained from the database of the CNSR study.[18] The additional costs of mechanical thrombectomy, including costs for devices, procedure and special periprocedural care, were estimated using the data from the EAST study and the Thrombolysis Implementation and Monitor of acute ischaemic Stroke in China (TIMS-CHINA, a national prospective registry of 1,440 acute ischaemic stroke patients with thrombolytic therapy with IV tPA recruited from 67 centres in China between May 2007 and April 2012.) study.[17 28] The additional costs of tPA treatment and occurrence of sICH were estimated using the data from the CNSR and the TIMS-CHINA studies. We did not include the indirect economic costs such as lost work productivity in the present study. All costs and utilities were discounted by 3% per year.[29]

Health States

Patients could undergo transitions between the three health states according to the functional outcome based on mRS: independent (mRS 0-2), dependent (mRS 3-5) or deceased (mRS 6).[7 30] At the end of each Markov cycle, the patients either remained in their current health state, attained a health state with greater disability due to recurrent stroke, or did not survived due to a recurrent stroke or a non-stroke cause (figure 1).

Outcome Assessment

Health outcomes were measured in terms of quality-adjusted life-years (QALYs), which were calculated by multiplying the length of life by utility scores derived from the literature.[9 31 32] The

For peer review only

utility scores of the different disability states after stroke were developed using the European quality of life scale (EQ-5D) along with the Chinese preference weights in a Chinese stroke population.[32] In the current model, the events of recurrent stroke and sICH were considered temporary health states unless they resulted in death. All patients who entered these health states were assumed to have a short-term disutility of 30 d for an event of recurrent stroke and only 14 d for an event of sICH.[9 31] The total economic costs were calculated by multiplying the number of patients in each state by the direct medical costs for that state. The incremental cost-effectiveness ratio (ICER) was calculated by dividing the difference of the costs by the difference in QALYs between the two treatment alternatives. We modelled the costs and QALYs gained over the short-term (1, 5, 6 years) and the long-term (30 years). The mechanical thrombectomy was considered cost-effective if the ICER was less than CNY 125,700 (3x GDP per capita of China in 2013,[26] US$ 19,300) per QALY gained. This willingness-to-pay threshold was recommended by the Commission on Macroeconomics and Health of World Health Organization.[29]

Sensitivity Analysis

A deterministic one-way sensitivity analysis that utilized varying probabilities, utilities and costs, was conducted in order to evaluate the uncertainty of the long-term (30 years) results of the model. The variation in these parameters was conducted once at a time at the plausible rages (table 1). In order to determine how much worse mechanical thrombectomy could have performed but still produced a cost-effective ICER, we constructed the two hypothetical worse outcomes for the performance of the mechanical thrombectomy. This was accomplished by setting the ORs of mRS 0-2 at day 90 for the mechanical thrombectomy treatment at the lower limits of 90% and 95% confidence intervals in the meta-analyses of the 9 trials. This represented

For peer review only

the unfavourable and worse unfavourable scenarios, respectively. For each outcome scenario, we further constructed four hypothetical cost scenarios by setting 10% increase or 10%, 25% and 50% decrease of the costs of mechanical thrombectomy.

Furthermore, a probabilistic sensitivity analysis was further undertaken in order to evaluate the stochastic uncertainty due to the simultaneous variability of the variables examined. It was conducted by using Monte Carlo simulation in Ersatz v1.3 (a bootstrap add-in for Microsoft Excel for Windows; EpiGear International Pty Ltd, Brisbane, Australia). We assumed that costs followed a lognormal distribution and that the probabilities and utilities followed a beta distribution. The simulation was run 10,000 times. The results were summarized using a scatter-plot and a cost-effectiveness acceptability curve.

Results

Base Case Analysis

Table 2 indicates the costs, outcomes and ICER for the mechanical thrombectomy treatment calculated in the short term (1, 5, 6 years) and in the long term (30 years). In the base-case scenario, for a 63-year-old patient with acute ischaemic stroke caused by proximal anterior circulation occlusion within 6 h after onset of stroke, mechanical thrombectomy would be cost-ineffective in the first five years, but become cost-effective from the sixth year onwards, using the threshold of CNY 125,700 (3x GDP per capita of China in 2013, US$ 19,300) as the willingness-to-pay per QALY. After 6 years, the mechanical thrombectomy gained 0.430 QALYs at an additional cost of CNY 48,940 (US$ 7,530), yielding an ICER of CNY 113,800 (US$ 17,510) per QALY gained. In the long term (30 years), mechanical thrombectomy gained

For peer review only

0.794 QALYs at an additional cost of CNY 50,000 (US$ 7,700), yielding an ICER of CNY 63,010 (US$ 9,690) per QALY gained.

Sensitivity Analysis

The results of the deterministic one-way sensitivity analysis for the ICER of the mechanical thrombectomy in the long term are presented in the tornado diagrams (figure 2). Overall, the ICER was most sensitive to the OR of the favourable functional outcome (mRS 0-2) at day 90, additional cost of mechanical thrombectomy and utility of mRS 0-2. In case of a decrease in the OR of mRS 0-2 at day 90 to 1.692, the ICER of the mechanical thrombectomy (CNY 87,123/QALY) was still within the threshold of the willingness-to-pay per QALY (CNY 125,700, 3x GDP per capita of China in 2013). In each hypothetical case scenario, the mechanical thrombectomy continued to produce a benefit in QALYs (online supplementary table 1). Even in the worst hypothetical scenario with worse unfavourable effect outcome and an increase of 10% price of the mechanical thrombectomy, this type of treatment continued to be cost-effective (ICER: CNY 95,839/QALY). In the hypothetical case scenario with a base case effect outcome and a decrease of 50% in the price of mechanical thrombectomy, this type of treatment could be highly cost-effective (ICER: CNY 30,995/QALY<CNY 41,900 [1x GDP per capita of China in 2013, US$ 6,400]/QALY).

Figure 3 indicates the results of the probabilistic sensitivity analysis in the long term with parameters of the model inputs presented in the online supplementary table 2. Among the 10,000 simulation runs, mechanical thrombectomy was cost-effective in 99.9% of the simulations at a willingness-to-pay threshold of CNY 125,700 (3x GDP per capita of China in 2013, US$ 19,300) per QALY. The online supplementary figure 1 indicates the cost-effectiveness acceptability curve of the mechanical thrombectomy.

For peer review only

Discussion

The present study indicated that mechanical thrombectomy with second generation devices for acute ischaemic stroke that was caused by proximal anterior circulation occlusion was cost-effective from the sixth year onwards in China. Each patient with acute ischaemic stroke treated with mechanical thrombectomy gained an ICER of CNY 63,010 per QALY in the long term, which was below 3x GDP per capita of China in 2013 (CNY 125,700). The study further demonstrated that the ICER was more sensitive to the OR of favourable functional outcome at day 90, additional cost of mechanical thrombectomy and the utility for patients with independence.

The present study indicated that mechanical thrombectomy treatment was cost-effective in low- and middle-income countries such as China, which is a similar conclusion to that completed in other high-income countries.[7-10] The lifetime gain of 0.794 QALYs with regard to the mechanical thrombectomy treatment for acute ischaemic stroke in this study was comparable to that reported in the high income countries (0.7 QALYs in the USA,[7] 1.05 QALYs in the UK[9] and 0.99 QALYs in Sweden[10]). The QALYs gain of mechanical thrombectomy treatment was relatively higher compared with that of the majority of the other treatments for stroke. For example, the lifetime QALYs gain was 0.42 for IV tPA treatment for acute ischaemic stroke within 4.5 h,[33] and 0.17 for clopidogrel for secondary prevention of stroke compared with aspirin.[34] This may be due to the large magnitude of the effect of the mechanical thrombectomy (OR 2.046).

Mechanical thrombectomy treatment with second generation devices has been accepted as the standard of care for acute ischaemic stroke patients caused by proximal anterior circulation

For peer review only - http://bmjopen.bmj.com/site/about/guidelines.xhtml

occlusions within 6 h after symptom onset.[35 36] However, economic costs of the mechanical thrombectomy treatment are extremely high, especially in low- and middle-income countries such as China, where people experience a higher incidence of stroke and higher prevalence of intracranial atherosclerosis compared to the Western countries.[37] The additional cost of the mechanical thrombectomy was approximately 5-fold higher compared with that noted for the one-time hospitalisation without tPA treatment and mechanical thrombectomy in China. This difference between the 2 aforementioned parameters was only 1.5-fold in the USA.[7 8] Previous studies indicated socioeconomic disparities with regard to the utilization of mechanical thrombectomy for acute ischaemic stroke and the patients with low income who were resident in remote areas exhibited lower rates of mechanical thrombectomy utilization.[38 39] The implementation of mechanical thrombectomy treatment was to-some-extent dependent on the cost-effectiveness of the technology, which is particularly significant for the clinical decision in the low- and middle-income countries. The present study supported the implementation of the mechanical thrombectomy treatment after acute ischaemic stroke in clinical practice in the low- and middle-income countries from the perspective of economics. The data may provide an important reference for the low income and/or remote areas in the Western countries.

Proximal large vessel atherosclerotic stenosis or occlusion accounts for 35–40% of all acute ischaemic strokes,[17] among which approximately 40% are admitted to the hospital within 6 h in China.[18] Therefore, approximately 14%-16% of ischaemic stroke patients were eligible for mechanical thrombectomy and might benefit from this procedure. However, the real-world implementation of endovascular thrombectomy treatment in low- and middle-income countries and areas may be restricted by the poor awareness of the public, poor infrastructure, inefficient systems, deficiency of specialists and the time points of patient entry to the hospital (within 6

For peer review only

hours), which may cause inequity for those who cannot receive the technology.[10] Thus, the education that is targeted to the public, hospital administration and governmental agencies should be improved so that users can fully understand the benefit and cost-effectiveness of thrombectomy.[40] Furthermore, the service system redesign is required to establish efficient care chains and workflow with coordination between neurointerventinalists and other departments. Additionally, high experience and skills are required to perform this advanced technology, while the ways of providing interventional treatment are non-unstandardized and diverse in low- and middle- income countries such as China.[40] Future studies should focus on the organization of standardized training for performing thrombectomy in order to compensate for the deficiency of intervention specialists in these countries and areas.

The current study has several limitations that should be considered when interpreting the results. First, our model focused on the impact of mechanical thrombectomy treatment on acute ischaemic stroke, and the health status and costs that were involved as a result of other causes, such as occurrence of intracranial haemorrhage and myocardial infarction, were not included in this model. Second, the costs of transfer to the hospitals doing mechanical thrombectomy were not included in this analysis. Third, functional improvement after rehabilitation was not considered in the model due to the lack of available data on the efficacy of rehabilitation. However, organized rehabilitation after stroke in China is poor.[41 42] Fourth, the current study arbitrarily assumed to some extent that the patients with independent state remaining alive after stroke recurrence were equally reallocated in the categories of independent and dependent states. However, this is not unprecedented in the modelling of cost-effectiveness analysis for stroke.[24] Finally, the efficacy of mechanical thrombectomy treatment was based on trials that were completed in high income countries with older age of participants compared with those reported

For peer review only

in China. In addition, the majority of the trials were terminated early and all were sponsored by the industry, which may have caused potential risk of bias, whereas some of the patients included in the trials did not receive IV tPA treatment. However, the current analysis included all data that were possibly available. These limitations would have led to under- or over- estimation of the true cost-effectiveness of mechanical thrombectomy treatment in low- and middle-income countries.

Conclusions

Mechanical thrombectomy with second generation devices for acute ischaemic stroke caused by proximal anterior circulation occlusion within 6 h after the onset of stroke was cost-effective in China. The current study supports the implementation of mechanical thrombectomy treatment after acute ischaemic stroke in clinical practice in low- and middle-income countries, and may also be a reference to the low income and/or remote areas in the developed countries. Additional medical resources that are related to mechanical thrombectomy should be allocated in these areas.

Contributors

YSP, YJW and YLW designed the study and drafted the manuscript. YSP, XLC and XCH: collected the data, performed the literature search and constructed the decision tree. XQZ, LPL and ZRM interpreted the data and revised the manuscript.

Funding

This work was supported by grants from the Ministry of Science and Technology of the People's Republic of China (2015BAI12B04, 2015BAI12B02, 2016YFC0901000, 2016YFC0901001,

For peer review only

2016YFC0901002), grants from Beijing Municipal Science and Technology Commission (Z151102003900000, Z151100003915117), and grants from Beijing Municipal Commission of Health and Family Planning (No.2016-1-2041, SML20150502).

Competing interests

None declared.

Data sharing statement

We, as the authors of this original research article, state that there are no additional, unpublished data available from this study.

For peer review only

References

1. Lees KR, Bluhmki E, von Kummer R, *et al*. Time to treatment with intravenous alteplase and outcome in stroke: an updated pooled analysis of ECASS, ATLANTIS, NINDS, and EPITHET trials. *Lancet* 2010;375:1695-703.
2. Berkhemer OA, Fransen PS, Beumer D, *et al*. A randomized trial of intraarterial treatment for acute ischemic stroke. *N Engl J Med* 2015;372:11-20.
3. Goyal M, Demchuk AM, Menon BK, *et al*. Randomized assessment of rapid endovascular treatment of ischemic stroke. *N Engl J Med* 2015;372:1019-30.
4. Saver JL, Goyal M, Bonafe A, *et al*. Stent-retriever thrombectomy after intravenous t-PA vs. t-PA alone in stroke. *N Engl J Med* 2015;372:2285-95.
5. Jovin TG, Chamorro A, Cobo E, *et al*. Thrombectomy within 8 hours after symptom onset in ischemic stroke. *N Engl J Med* 2015;372:2296-306.
6. Campbell BC, Mitchell PJ, Kleinig TJ, *et al*. Endovascular therapy for ischemic stroke with perfusion-imaging selection. *N Engl J Med* 2015;372:1009-18.
7. Leppert MH, Campbell JD, Simpson JR, Burke JF. Cost-Effectiveness of Intra-Arterial Treatment as an Adjunct to Intravenous Tissue-Type Plasminogen Activator for Acute Ischemic Stroke. *Stroke* 2015;46:1870-6.
8. Kunz WG, Hunink MG, Sommer WH, *et al*. Cost-Effectiveness of Endovascular Stroke Therapy: A Patient Subgroup Analysis From a US Healthcare Perspective. *Stroke* 2016;47:2797-804.
9. Ganesalingam J, Pizzo E, Morris S, Sunderland T, Ames D, Lobotesis K. Cost-Utility Analysis of Mechanical Thrombectomy Using Stent Retrievers in Acute Ischemic Stroke. *Stroke* 2015;46:2591-8.

For peer review only

10. Aronsson M, Persson J, Blomstrand C, Wester P, Levin LÅ. Cost-effectiveness of endovascular thrombectomy in patients with acute ischemic stroke. *Neurology* 2016;86:1053-9.

11. Lobotesis K, Veltkamp R, Carpenter IH, Claxton LM, Saver JL, Hodgson R. Cost-effectiveness of Stent-retriever Thrombectomy in combination with IV t-PA Compared with IV t-PA Alone for Acute Ischemic Stroke in the UK. *J Med Econ* 2016:1-37.

12. Steen Carlsson K, Andsberg G, Petersson J, Norrving B. Long-term cost-effectiveness of thrombectomy for acute ischaemic stroke in real life: An analysis based on data from the Swedish Stroke Register (Riksstroke). *Int J Stroke* 2017:1747493017701154.

13. de Andrés-Nogales F, Álvarez M, de Miquel MÁ, et al. Cost-effectiveness of mechanical thrombectomy using stent retriever after intravenous tissue plasminogen activator compared with intravenous tissue plasminogen activator alone in the treatment of acute ischaemic stroke due to large vessel occlusion in Spain. *Eur Stroke J* 2017;2:272-84.

14. Shireman TI, Wang K, Saver JL, et al. Cost-Effectiveness of Solitaire Stent Retriever Thrombectomy for Acute Ischemic Stroke: Results From the SWIFT-PRIME Trial (Solitaire With the Intention for Thrombectomy as Primary Endovascular Treatment for Acute Ischemic Stroke). *Stroke* 2017;48:379-87.

15. Xie X, Lambrinos A, Chan B, et al. Mechanical thrombectomy in patients with acute ischemic stroke: a cost-utility analysis. *CMAJ Open* 2016;4:E316-25.

16. Yang G, Wang Y, Zeng Y, *et al*. Rapid health transition in China, 1990-2010: findings from the Global Burden of Disease Study 2010. *Lancet* 2013;381:1987-2015.

For peer review only

17. Miao Z, Huo X, Gao F, *et al*. Endovascular therapy for Acute ischemic Stroke Trial (EAST): study protocol for a prospective, multicentre control trial in China. *Stroke Vasc Neurol* 2016;1:44-51.

18. Wang Y, Cui L, Ji X, *et al*. The China National Stroke Registry for patients with acute cerebrovascular events: design, rationale, and baseline patient characteristics. *Int J Stroke* 2011;6:355-61.

19. Bracard S, Ducrocq X, Mas JL, *et al*. Mechanical thrombectomy after intravenous alteplase versus alteplase alone after stroke (THRACE): a randomised controlled trial. *Lancet Neurol* 2016;15:1138-47.

20. Mocco J, Zaidat OO, von Kummer R, *et al*. Aspiration Thrombectomy After Intravenous Alteplase Versus Intravenous Alteplase Alone. *Stroke* 2016;47:2331-8.

21. Muir KW, Ford GA, Messow CM, *et al*. Endovascular therapy for acute ischaemic stroke: the Pragmatic Ischaemic Stroke Thrombectomy Evaluation (PISTE) randomised, controlled trial. *J Neurol Neurosurg Psychiatry* 2017;88:38-44.

22. Khoury NN, Darsaut TE, Ghostine J, *et al*. Endovascular thrombectomy and medical therapy versus medical therapy alone in acute stroke: A randomized care trial. *J Neuroradiol* 2017;44:198-202.

23. Pennlert J, Eriksson M, Carlberg B, Wiklund PG. Long-term risk and predictors of recurrent stroke beyond the acute phase. *Stroke* 2014;45:1839-41.

24. Tung CE, Win SS, Lansberg MG. Cost-effectiveness of tissue-type plasminogen activator in the 3- to 4.5-hour time window for acute ischemic stroke. *Stroke* 2011;42:2257-62.

For peer review only

25. National Bureau of Statistics of China. The 2010 population census of the peaple's republic of china. Websit: http://www.stats.gov.cn/tjsj/pcsj/rkpc/6rp/indexch.htm (in Chinese). [Accessed Feb 25, 2016].

26. Ministry of Health of the People's Republic of China. China Health Statistics Yearbook 2014. Peking Union Medical College Press. Beijing (2014) (in Chinese).

27. Samsa GP, Reutter RA, Parmigiani G, *et al*. Performing cost-effectiveness analysis by integrating randomized trial data with a comprehensive decision model: application to treatment of acute ischemic stroke. *J Clin Epidemiol* 1999;52:259-71.

28. Liao XL, Wang CX, Wang YL, *et al*. Implementation and outcome of thrombolysis with alteplase 3 to 4.5 h after acute stroke in Chinese patients. *CNS Neurosci Ther* 2013;19:43-7.

29. Pan Y, Wang A, Liu G, *et al*. Cost-effectiveness of clopidogrel-aspirin versus aspirin alone for acute transient ischemic attack and minor stroke. *J Am Heart Assoc* 2014;3:e000912.

30. Kim AS, Nguyen-Huynh M, Johnston SC. A cost-utility analysis of mechanical thrombectomy as an adjunct to intravenous tissue-type plasminogen activator for acute large-vessel ischemic stroke. *Stroke* 2011;42:2013-8.

31. Lee S, Anglade MW, Pham D, Pisacane R, Kluger J, Coleman CI. Cost-effectiveness of rivaroxaban compared to warfarin for stroke prevention in atrial fibrillation. *Am J Cardiol* 2012;110:845-51.

32. Wang YL, Pan YS, Zhao XQ, *et al*. Recurrent stroke was associated with poor quality of life in patients with transient ischemic attack or minor stroke: finding from the CHANCE trial. *CNS Neurosci Ther* 2014;20:1029-35.

33. Pan Y, Chen Q, Zhao X, *et al*. Cost-Effectiveness of Thrombolysis within 4.5 Hours of Acute Ischemic Stroke in China. *PLoS One* 2014;9:e110525.

For peer review only

34. Schleinitz MD, Weiss JP, Owens DK. Clopidogrel versus aspirin for secondary prophylaxis of vascular events: a cost-effectiveness analysis. *Am J Med* 2004;116:797-806.

35. Powers WJ, Derdeyn CP, Biller J, *et al*. 2015 American Heart Association/American Stroke Association Focused Update of the 2013 Guidelines for the Early Management of Patients With Acute Ischemic Stroke Regarding Endovascular Treatment: A Guideline for Healthcare Professionals From the American Heart Association/American Stroke Association. *Stroke* 2015;46:3020-35.

36. Miao Z, Wang Y. Chinese guidelines for endovascular treatment for acute ischemic stroke 2015.(in Chinese). *Chin J Stroke* 2015;10:590-606.

37. Wang Y, Zhao X, Liu L, *et al*. Prevalence and outcomes of symptomatic intracranial large artery stenoses and occlusions in China: the Chinese Intracranial Atherosclerosis (CICAS) Study. *Stroke* 2014;45:663-9.

38. Brinjikji W, Rabinstein AA, Cloft HJ. Socioeconomic disparities in the utilization of mechanical thrombectomy for acute ischemic stroke. *J Stroke Cerebrovasc Dis* 2014;23:979-84.

39. Pérez de la Ossa N, Abilleira S, Dorado L, *et al*. Access to Endovascular Treatment in Remote Areas: Analysis of the Reperfusion Treatment Registry of Catalonia. *Stroke* 2016;47:1381-4.

40. Miao Z. New era of stroke therapy and new challenges. *Stroke Vasc Neurol* 2016;1:6-7.

41. Li AD, Huang ZQ, Liu HT, Deng YT, Xiao JW, Liang JW. Knowledge, Attitude and Behavior for Stroke and Rehabilitation in Stroke Patients and Relatives.(in Chinese). *Chin J Rehabil Theory Pract* 2009;15:252-4.

For peer review only

42. Gao YL, Chen LD, Tao J, Chen SM. Investigation on the investment and popularization of rehabilitation from cerebral apoplexy: in 2 communities of Xiamen City.(in Chinese). *Chin J Convalescent Med* 2009;18:472-4.

For peer review only

Figure Legends

Figure 1. Decision tree and Markov state transition model. A patient with an acute ischaemic stroke with anterior circulation occlusion entered the model at 63 years old receiving either intravenous tissue-type plasminogen activator (IV tPA) with or without mechanical thrombectomy, and transited between health states until death or 30 years. Patients may remain in the same health state, move to a state of equal or greater disability after recurrent stroke, or die. Only transition from dependent state (modified Rankin Scale [mRS] 3-5) was illustrated in the figure. M indicates Markov node; sICH, symptomatic intracerebral haemorrhage.

Figure 2. One-way sensitivity analysis on incremental cost-effectiveness ratio (ICER) gained in the long term (30 years) by mechanical thrombectomy. All model input parameters were analysed, and only ten parameters with the highest relative effects on ICER are displayed. Base-case scenario of ICER is CNY 63,010 per quality-adjusted life-year (QALY) gained.

Figure 3. Scatterplot of the result of probabilistic sensitivity analysis in the long term (30 years). Each point represents a simulation. The dark square represents the base case (0.794 QALYs gained at an incremental cost of CNY 50,000). The solid line represents the willingness-to-pay threshold of CNY 125,700 per QALY. The dashed line represents CNY 41,900 per QALY. Points to the right of the solid line are considered cost-effective.

For peer review only

Tables

Table 1. Base-case and Plausible Ranges of Model Inputs

Model Input	Base Case	Range	Reference
**Efficacy and safety outcome inputs**			[2-6 19-22]
Proportion of outcomes at 90 days in IV tPA group			
mRS 0-2	0.325	0.258-0.392	
Death (mRS 6)	0.168	0.131-0.205	
sICH	0.058	0.035-0.095	
Odds ratio at 90 days			
mRS 0-2	2.046	1.692-2.474	
Death (mRS 6)	0.871	0.684-1.109	
sICH	0.965	0.665-1.399	
**Probabilities inputs**			
Proportion of patients received mechanical thrombectomy	0.861	0.839-0.883	[2-6 19-22]
Proportion of patients arrived within 4.5 hours	0.854	0.839-0.869	CNSR
Recurrent rate of stroke (per patient year)			CNSR
mRS 0-2	0.1026	0.0961-0.1093	
mRS 3-5	0.1418	0.1303-0.1534	
Relative risk of stroke recurrence per life year	1.03	1.02-1.04	[23]
Death with recurrent stroke	0.2101	0.1887-0.2316	CNSR
Age specific non-stroke death rate *	0.0089-0.1653		[25 26]

For peer review only

Hazard ratio of non-stroke death for mRS 3-5	1.78	1.43-2.14	[27]
**Cost inputs (2013 Chinese Yuan Renminbi)**			
Additional costs of mechanical thrombectomy	60821	52314-70311	EAST
Additional costs of IV tPA treatment	11179	10555-11829	CNSR, TIMS-CHINA
Additional costs of sICH	2374	2249-2504	TIMS-CHINA
One-time hospitalisation costs			CNSR
mRS 0-2	10055	9907-10205	
mRS 3-5	13729	13428-14035	
mRS 6	11121	10219-12081	
Annual post-hospitalisation costs			CNSR
mRS 0-2	7385	7156-7619	
mRS 3-5	11350	10730-11996	
**Utility inputs**			
mRS 0-2	0.76	0.69-0.82	[32]
mRS 3-5	0.21	0.17-0.26	[32]
Death (mRS 6)	0	0.00-0.00	[32]
Recurrent stroke	0.34	0.32-0.36	[9]
sICH	0.84	0.72-1.0	[31]
**Discount rate inputs**			
Costs	0.03	0.03-0.08	[29]
Outcomes	0.03	±20%	[29]

For peer review only

All costs were converted to 2013 Chinese Yuan Renminbi by using the medical care component of consumer price index; to convert CNY to US dollars, divide by 6.5.

IV tPA, intravenous tissue plasminogen activator; mRS, modified Rankin Scale; sICH, symptomatic intracerebral haemorrhage; CNSR, China National Stroke Registry; EAST, Endovascular therapy for Acute ischaemic Stroke Trial; TIMS-CHINA, Thrombolysis Implementation and Monitor of acute ischaemic Stroke in China.

* Age specific non-stroke death rate: only the number of 63 years old (0.0089) and 93 years old (0.1653) are presented.

For peer review only

Table 2. Costs and Outcomes in Base-case Analysis

Time horizon	Treat strategy	QALYs	Cost (CNY)	ICER (CNY/QALY)
1 year	IV tPA alone	0.326	27,220	-
	Mechanical thrombectomy + IV tPA	0.405	77,700	638,987
5 years	IV tPA alone	1.392	58,590	-
	Mechanical thrombectomy + IV tPA	1.765	107,710	131,689
6 years	IV tPA alone	1.599	65,230	-
	Mechanical thrombectomy + IV tPA	2.029	114,170	113,814
30 years	IV tPA alone	2.979	117,940	-
	Mechanical thrombectomy + IV tPA	3.773	167,970	63,010

IV tPA, intravenous tissue plasminogen activator; QALY, quality-adjusted life-year; ICER, incremental cost-effectiveness ratio.

For peer review only - http://bmjopen.bmj.com/site/about/guidelines.xhtml

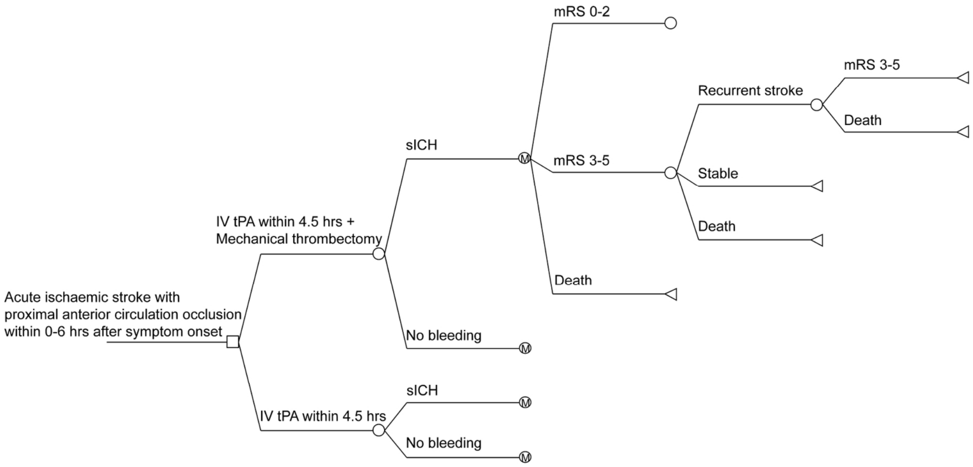

Figure 1. Decision tree and Markov state transition model. A patient with an acute ischaemic stroke with anterior circulation occlusion entered the model at 63 years old receiving either intravenous tissue-type plasminogen activator (IV tPA) with or without mechanical thrombectomy, and transited between health states until death or 30 years. Patients may remain in the same health state, move to a state of equal or greater disability after recurrent stroke, or die. Only transition from dependent state (modified Rankin Scale [mRS] 3-5) was illustrated in the figure. M indicates Markov node; sICH, symptomatic intracerebral haemorrhage.

84x41mm (600 x 600 DPI)

For peer review only

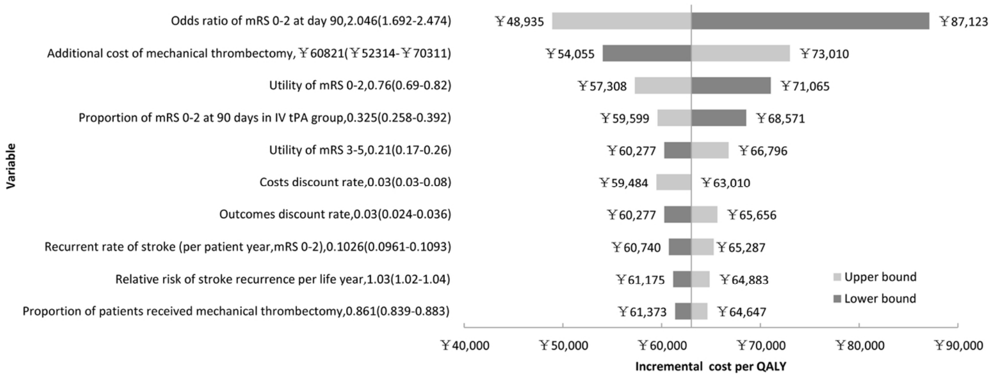

Figure 2. One-way sensitivity analysis on incremental cost-effectiveness ratio (ICER) gained in the long term (30 years) by mechanical thrombectomy. All model input parameters were analysed, and only ten parameters with the highest relative effects on ICER are displayed. Base-case scenario of ICER is CNY 63,010 per quality-adjusted life-year (QALY) gained.

66x25mm (600 x 600 DPI)

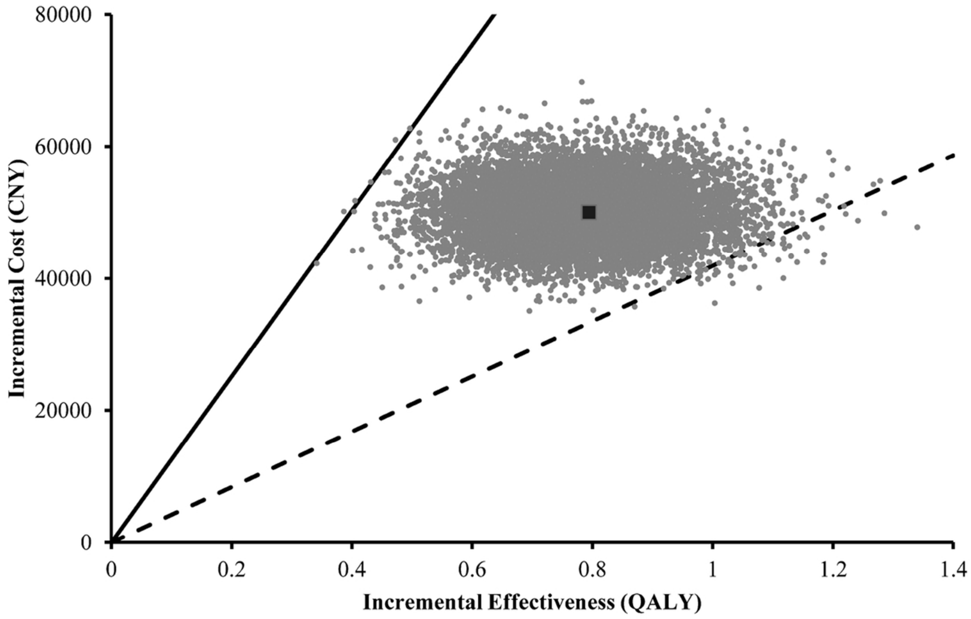

Figure 3. Scatterplot of the result of probabilistic sensitivity analysis in the long term (30 years). Each point represents a simulation. The dark square represents the base case (0.794 QALYs gained at an incremental cost of CNY 50,000). The solid line represents the willingness-to-pay threshold of CNY 125,700 per QALY. The dashed line represents CNY 41,900 per QALY. Points to the right of the solid line are considered cost-effective.

54x36mm (600 x 600 DPI)

ONLINE SUPPLEMENTARY MATERIALS

Online supplementary table 1. Hypothetical worse performance and costs of mechanical thrombectomy, ICER (CNY/QALY)

	Outcome of mechanical thrombectomy		
**Additional cost of mechanical thrombectomy**	**Base Case (OR=2.046)***	**Unfavourable (OR=1.745)**	**Worse unfavourable (OR=1.692)**
+10%, CNY 66903	69,421	90,340	95,839
Base case, CNY 60821	63,010	82,120	87,123
-10%, CNY 54739	56,612	73,883	78,425
-25%, CNY 45616	47,003	61,553	65,360
-50%, CNY 30410	30,995	40,987	43,596

ICER, incremental cost-effectiveness ratio; QALY, quality-adjusted life-year; OR, Odds ratio.

* OR: OR of modified Rankin Scale 0-2 at day 90 for mechanical thrombectomy.

For peer review only

Online supplementary table 2. Distributions and parameters used in model inputs

Model Input	Distribution	α	β	mean	standard error
**Efficacy and safety outcome inputs**					
Proportion of outcomes at 90 days in IV tPA group					
mRS 0-2	beta	60.7	126.0		
Death (mRS 6)	beta	65.7	325.5		
sICH	beta	13.5	218.7		
Odds ratio at 90 days					
mRS 0-2	lognormal*			2.046	0.097
Death (mRS 6)	lognormal*			0.871	0.123
sICH	lognormal*			0.965	0.190
**Probabilities inputs**					
Proportion of patients received mechanical thrombectomy	beta	817.0	131.9		
Proportion of patients arrived within 4.5 hours	beta	1817.2	310.7		
Recurrent rate of stroke (per patient year)					
mRS 0-2	beta	833.0	7286.0		
mRS 3-5	beta	496.8	3006.6		
Relative risk of stroke recurrence per life year	lognormal*			1.03	0.005
Death with recurrent stroke	beta	290.9	1093.7		
Hazard ratio of non-stroke death for mRS 3-5	lognormal*			1.78	0.103
**Cost inputs (2013 Chinese Yuan Renminbi)**					
Additional costs of mechanical thrombectomy	lognormal			60821	4594
Additional costs of IV tPA treatment	lognormal			11179	325
Additional costs of sICH	lognormal			2374	65
One-time hospitalisation costs					
mRS 0-2	lognormal			10055	76
mRS 3-5	lognormal			13729	155
mRS 6	lognormal			11121	475
Annual post-hospitalisation costs					
mRS 0-2	lognormal			7385	118
mRS 3-5	lognormal			11350	323
**Utility inputs**					
mRS 0-2	beta	125.3	39.6		
mRS 3-5	beta	65.9	247.8		
Recurrent stroke	beta	732.4	1421.7		
sICH	beta	21.3	4.1		
**Discount rate inputs**					
Costs	beta	5.3	172.5		
Outcomes	beta	93.1	3011.2		

IV tPA, intravenous tissue plasminogen activator; mRS, modified Rankin Scale; sICH, symptomatic intracerebral haemorrhage.
*Correction is implemented in the Ersatz function ErRelativeRisk(RR, SE[ln(RR)]).

For peer review only

Online supplementary figure 1. Cost-effectiveness acceptability curve. The curve presents the probability that mechanical thrombectomy within 6 hours to be cost-effective against willingness-to-pay threshold. The solid line represents the willingness-to-pay threshold of CNY 125,700 per QALY. The dashed line represents CNY 41,900 per QALY.

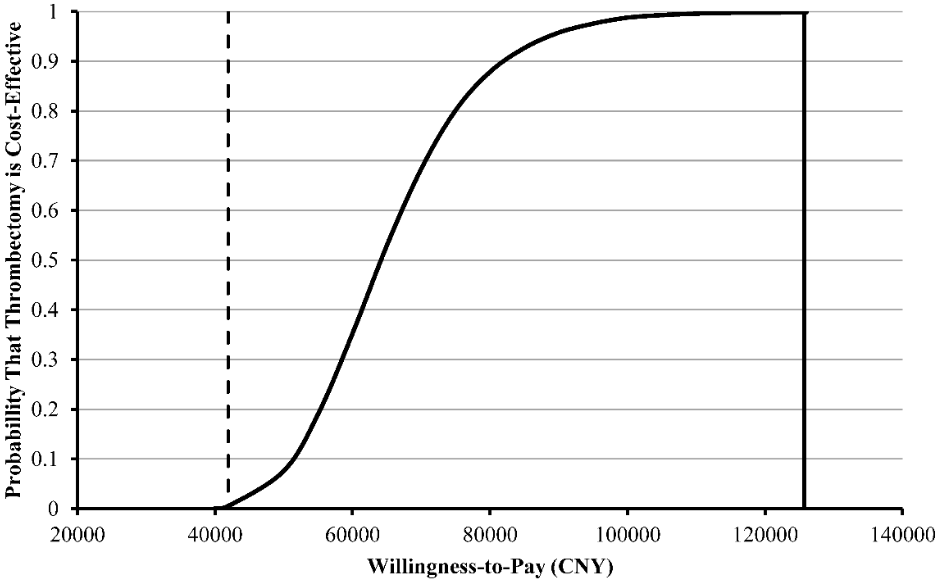

Table

Table 1| **CHEERS checklist—Items to include when reporting economic evaluations of health interventions**

Section/item	Item No	Recommendation	Reported on page No/ line No
**Title and abstract**			
Title	1	Identify the study as an economic evaluation or use more specific terms such as "cost-effectiveness analysis", and describe the interventions compared.	Title Page
Abstract	2	Provide a structured summary of objectives, perspective, setting, methods (including study design and inputs), results (including base case and uncertainty analyses), and conclusions.	Page 1
**Introduction**			
Background and objectives	3	Provide an explicit statement of the broader context for the study.	Page 4
		Present the study question and its relevance for health policy or practice decisions.	Page 4
**Methods**			
Target population and subgroups	4	Describe characteristics of the base case population and subgroups analysed, including why they were chosen.	Page 5
Setting and location	5	State relevant aspects of the system(s) in which the decision(s) need(s) to be made.	Page 5
Study perspective	6	Describe the perspective of the study and relate this to the costs being evaluated.	Page 6
Comparators	7	Describe the interventions or strategies being compared and state why they were chosen.	Page 5
Time horizon	8	State the time horizon(s) over which costs and consequences are being evaluated and say why appropriate.	Page 9
Discount rate	9	Report the choice of discount rate(s) used for costs and outcomes and say why appropriate.	Page 8
Choice of health outcomes	10	Describe what outcomes were used as the measure(s) of benefit in the evaluation and their relevance for the type of analysis performed.	Page 8
Measurement of effectiveness	11a	*Single study-based estimates:* Describe fully the design features of the single effectiveness study and why the single study was a sufficient source of clinical effectiveness data.	
	11b	*Synthesis-based estimates:* Describe fully the methods used for identification of included studies and synthesis of clinical effectiveness data.	Page 6-7
Measurement and valuation of preference based outcomes	12	If applicable, describe the population and methods used to elicit preferences for outcomes.	Page 8
Estimating resources and costs	13a	*Single study-based economic evaluation:* Describe approaches used to estimate resource use associated with the alternative interventions. Describe primary or secondary research methods for valuing each resource item in terms of its unit cost. Describe any adjustments made to approximate to opportunity costs.	Page 8
	13b	*Model-based economic evaluation:* Describe approaches and data sources used to estimate resource use associated with model health states. Describe primary or secondary research methods for valuing each resource item in terms of its unit cost. Describe any adjustments made to approximate to opportunity costs.	
Currency, price date, and conversion	14	Report the dates of the estimated resource quantities and unit costs. Describe methods for adjusting estimated unit costs to the year of reported costs if necessary. Describe methods for converting costs into a common currency base and the exchange rate.	Page 8
Choice of model	15	Describe and give reasons for the specific type of decision-analytical model used. Providing a figure to show model structure is strongly recommended.	Page 5
Assumptions	16	Describe all structural or other assumptions underpinning the decision-analytical model.	Page 5
Analytical methods	17	Describe all analytical methods supporting the evaluation. This could include methods for dealing with skewed, missing, or censored data; extrapolation methods; methods for pooling data; approaches to validate or make adjustments (such as half cycle corrections) to a model; and methods for handling population heterogeneity and uncertainty.	Page 8-9
**Results**			
Study parameters	18	Report the values, ranges, references, and, if used, probability distributions for all parameters. Report reasons or sources for distributions used to represent uncertainty where appropriate. Providing a table to show the input values is strongly recommended.	Table 1
Incremental costs and outcomes	19	For each intervention, report mean values for the main categories of estimated costs and outcomes of interest, as well as mean differences between the comparator groups. If applicable, report incremental cost-effectiveness ratios.	Page 10,Table 2
Characterising uncertainty	20a	*Single study-based economic evaluation:* Describe the effects of sampling uncertainty for the estimated incremental cost and incremental effectiveness parameters, together with the impact of methodological assumptions (such as discount rate, study perspective).	Page 11

For peer review only

No commercial reuse: See rights and reprints http://www.bmj.com/permissions Subscribe: http://www.bmj.com/subscribe

BMJ 2013;346:f1049 doi: 10.1136/bmj.f1049 (Published 25 March 2013)

(continued)

Section/item	Item No	Recommendation	Reported on page No/ line No
	20b	*Model-based economic evaluation:* Describe the effects on the results of uncertainty for all input parameters, and uncertainty related to the structure of the model and assumptions.	Page 11
Characterising heterogeneity	21	If applicable, report differences in costs, outcomes, or cost-effectiveness that can be explained by variations between subgroups of patients with different baseline characteristics or other observed variability in effects that are not reducible by more information.	Page 11
**Discussion**			
Study findings, limitations, generalisability, and current knowledge	22	Summarise key study findings and describe how they support the conclusions reached. Discuss limitations and the generalisability of the findings and how the findings fit with current knowledge.	Page 12-14
**Other**			
Source of funding	23	Describe how the study was funded and the role of the funder in the identification, design, conduct, and reporting of the analysis. Describe other non-monetary sources of support.	Page 15
Conflicts of interest	24	Describe any potential for conflict of interest of study contributors in accordance with journal policy. In the absence of a journal policy, we recommend authors comply with International Committee of Medical Journal Editors recommendations.	Page 16

For consistency, the CHEERS statement checklist format is based on the format of the CONSORT statement checklist

For peer review only

For peer review only - http://bmjopen.bmj.com/site/about/guidelines.xhtml

No commercial reuse: See rights and reprints http://www.bmj.com/permissions Subscribe: http://www.bmj.com/subscribe
